# Comparative immunogenicity and protective efficacy of BECC438 and BECC470 adjuvants in a vaccine formulation against *Pseudomonas aeruginosa*

**DOI:** 10.3389/fimmu.2026.1908082

**Published:** 2026-09-03

**Authors:** Prolay Halder, Debaki Ranjan Howlader, Satabdi Biswas, Risha Haldar, Suhrid Maiti, Sayan Das, Zackary K. Dietz, Ti Lu, Sean K. Whittier, Robert K. Ernst, William D. Picking, Wendy L. Picking

**Affiliations:** 1 Bond Life Sciences Center, University of Missouri, Columbia, MO, United States; 2 Department of Pathobiology & Integrative Biomedical Sciences, University of Missouri, Columbia, MO, United States; 3 Department of Microbial Pathogenesis, University of Maryland, Baltimore, MD, United States

**Keywords:** BECC438, BECC470, humoral immunity, intramuscular immunization, intranasal immunization, mucosal immunity, *Pseudomonas aeruginosa*, vaccine adjuvants

## Abstract

The development of effective vaccines against the opportunistic pathogen *Pseudomonas aeruginosa* (Pa) remains a critical public health priority for susceptible individuals due, in part, to the pathogen’s intrinsic resistance mechanisms. This study evaluates the comparative immunogenicity and protective efficacy of two novel lipid A mimetic adjuvants, BECC438 (BECC438s or BECC438b) and BECC470 (BECC470s or BECC470b), in a subunit vaccine (L-PaF – a genetic fusion of Pa type III secretion system proteins PcrV and PopB with LTA1, the active moiety of heat-labile enterotoxin from enterotoxigenic *E. coli*) formulated into an oil-in-water (o/w) emulsion to target Pa. BECC438b and BECC470b are biologically derived lipid A analogues produced using a Bacterial Enzymatic Combinatorial Chemistry (BECC) platform. In contrast, BECC438s and BECC470s are chemically synthesized counterparts designed to achieve high purity, eliminating congener species that could interfere with selective Toll-like receptor 4 (TLR4) activation while minimizing potential reactogenicity. Vaccine formulations incorporating BECC438 or BECC470 with Pa antigens were administered to mice via intranasal (IN) or intramuscular (IM) routes, followed by assessment of immune responses and lung burden post-challenge with Pa. IN immunization with BECC438s or BECC438b induced robust antigen-specific IgG and IgA responses, with a balanced IgG1/IgG3 profile that is indicative of mixed Th1/Th2 polarization, however, IgG1 remained the predominant antibody subclass. BECC438s conferred the highest protection against Pa challenge by reducing bacterial burden and improving immune responses following IN immunization. Moreover, L-PaF/ME/BECC438s delivered IN provided superior protection when compared to IM immunization. Serum antibody titers and pre-challenged or post-challenged cytokine responses were directly correlated with bacterial clearance upon Pa pulmonary challenge. These findings suggest that BECC438 offers enhanced mucosal and systemic immunity over BECC470 in this infection model, making it a promising candidate for inclusion in mucosal next-generation Pa vaccines. Overall, this comparative analysis highlights the potential of BECC derived adjuvants to improve vaccine performance against multidrug-resistant pathogens like Pa following IN immunization. Further studies are warranted to evaluate their translational potential in human populations.

## Introduction

*Pseudomonas aeruginosa* (Pa) is a Gram-negative opportunistic pathogen that poses a significant threat to immunocompromised individuals, particularly those with cystic fibrosis (CF), burn wounds, or ventilator-associated pneumonia (1, 2). Its intrinsic resistance to antibiotics, coupled with its ability to acquire new resistance mechanisms, has rendered many conventional treatments ineffective, prompting the World Health Organization (WHO) to classify Pa as a critical priority pathogen for the development of new antimicrobial strategies (3). From 2017 to 2018, hospital-onset Pa infections in U.S. acute care hospitals declined by 15% (4); however, the global burden surged in 2019 with over 500,000 deaths attributed to Pa, including more than 250,000 deaths linked to antimicrobial resistance (AMR). In 2020, during the COVID-19 pandemic, increased ventilator use and prolonged hospital stays led to a 29% increase in infections and 44% rise in carbapenem-resistant Pa (CRPA) infections compared to 2019 (4). By 2021–2022, multidrug-resistant (MDR) Pa prevalence in Asia and Africa reached 46%, with 19.6% of the isolates classified as extensively drug-resistant (XDR). Resistance to β-lactams exceeded 84%, while polymyxin resistance remained low (<6%) (5). In 2024, the WHO reaffirmed Pa as a critical priority pathogen in its updated bacterial priority pathogens list. The report emphasized Pa’s contributions in ventilator-associated pneumonia, bloodstream infections, and its resistance to last-resort antibiotics like carbapenems and colistin (6). The global spread of XDR strains underscores the urgent need for novel therapeutics, vaccines, and robust surveillance systems.

Pa is a major cause of chronic and acute pulmonary infections, particularly in individuals with underlying respiratory conditions. In patients with CF, ~50% of adolescents are chronically colonized by their late teens (1). The CF lung environment is characterized by thick mucus, impaired mucociliary clearance, and chronic inflammation that supports persistent Pa colonization and biofilm formation, leading to progressive lung damage and reduced life expectancy (7, 8). Beyond CF, Pa colonization also is associated with accelerated lung function decline, and increased mortality in other at-risk patients. For example, in patients with chronic obstructive pulmonary disease (COPD) and non-CF bronchiectasis (nCFB) (9, 10), Pa is often linked to higher rates of pneumonia and acquired antibiotic resistance (11). Pa infections are also associated with healthcare exposure and aging itself is a major independent risk factor for acute, life-threatening Pa infections (12). Collectively, these findings underscore the clinical importance of Pa across a spectrum of pulmonary conditions and age groups.

Despite extensive research, there are currently no licensed vaccines to prevent Pa infections, though several candidates are in development (12, 13). The genetic diversity and antigenic variability of Pa limit the efficacy of many vaccines targeting strain-specific components. In contrast, the highly conserved type III secretion system (T3SS), particularly surface PcrV and PopB, offers promising antigenic targets with >96% sequence conservation (14). These proteins are essential for T3SS function, and mutations typically attenuate virulence, minimizing vaccine escape (15). Previously, we have shown that intranasal (IN) administration of PcrV and PopB with dmLT, double-mutant labile toxin from enterotoxigenic *E. coli*, protected mice from pulmonary Pa challenge (16). dmLT is an adjuvant that triggers an immune response characterized by increased IgG2a, IgA, and IL-17A at mucosal sites (17). A PcrV-PopB fusion protein (PaF) was subsequently fused with LTA1, the active moiety of dmLT, to create the self‑adjuvanting L-PaF in a move to further improve its immunogenic potential. We have previously demonstrated that L-PaF induces strong IgG, IgA, IL-17A, and opsonophagocytic killing responses, allowing for lung clearance in animal models that is independent of serotype (18). These findings support the potential for L-PaF to be a broadly protective mucosal vaccine, particularly for high-risk groups like those with CF or ventilator-associated pneumonia (19, 20).

Adjuvants play a pivotal role in enhancing vaccine efficacy by modulating the magnitude and quality of the immune response. Traditional adjuvants, such as aluminum salts, primarily induce Th2-biased responses, which may be suboptimal for pathogens like Pa that require robust Th1 and Th17 immunity for effective clearance (21). The respiratory mucosa presents unique challenges for vaccine delivery, as mucociliary clearance mechanisms and the presence of a thick mucus layer can rapidly remove or trap vaccine antigens, thereby limiting antigen uptake and retention at the site of immunization (22). These barriers highlight the need for adjuvants that actively stimulate local innate immune responses and promote engagement of mucosal dendritic cell networks. In recent years, novel adjuvant platforms including toll-like receptor (TLR) agonists that mimic pathogen-associated molecular patterns (PAMPs) and activate innate immunity, have been developed. Among these, the Bacterial Enzymatic Combinatorial Chemistry (BECC) platform has gained attention for its ability to generate novel lipid A analogues that selectively engage the MD-2/TLR4 receptor complex. BECC438b and BECC470b are two engineered lipid A derivatives designed to retain immunostimulatory properties while minimizing endotoxicity (23). These molecules act as partial TLR4 agonists, promoting balanced Th1/Th2 responses and enhancing antigen-specific antibody production without triggering excessive inflammation (24). They are also attractive because they can enhance dendritic cell activation and antigen presentation and support the development of protective adaptive immunity while maintaining a favorable safety profile (25). Preclinical studies have demonstrated the potential of BECC438b and BECC470b as adjuvants in bacterial and viral subunit vaccines (23, 26). Notably, BECC470b has shown superior performance in aged mouse models, suggesting its utility in vulnerable immunosenescent populations (26). More recently, highly pure synthetic forms of these adjuvants (BECC438s and BECC470s) have been generated and found to have physical properties that are distinct from those of their biologically-derived counterparts (BECC438b and BECC470b) (27). Despite these promising findings, the comparative immunogenicity and protective efficacy of BECC438 (b and s) versus BECC470 (b and s) in the context of Pa vaccination remain unexplored. This study aims to assess the immunogenic profiles and protective outcomes of vaccine formulations incorporating BECC438 and BECC470 in a murine pulmonary model of Pa infection. We hypothesize that both adjuvants will enhance antigen-specific immune responses but may differ in their ability to induce optimal protective immunity and cytokine polarization. By dissecting these differences, we seek to inform the selection of optimal adjuvant candidates for future clinical development against Pa and other multidrug-resistant pathogens.

## Materials and methods

### Materials

BECC438b and BECC470b were prepared by extraction from *Yersinia pestis* after introducing lipid-A modifying enzymes (28). To overcome heterogeneity in the biological production of BECC438b and BECC470b, the chemically synthesized forms, BECC438s and BECC470s, were produced (27). They have been previously shown to be non-toxic in rodent (29, 30). Squalene was purchased from Echelon Biosciences (Salt Lake City, UT). Pa mPa08-31, provided by Dr. Susan E. Birket, is a clinical strain isolated from a CF patient at the University of Alabama-Birmingham. Chromatography columns were from GE Healthcare (Piscataway, NJ, USA). All other reagents and chemicals were from Millipore-Sigma Chemical Co. (St. Louis, MO) or Thermo-Fisher Scientific (Waltham, MA) and were chemical-grade or higher.

### Protein preparation

L-PaF was produced as previously described with minor modifications (16, 31). Briefly, *E. coli* HMS174(DE3) harboring *ltA1-pcrV-popB*/*histag-pcrH*//pACYCDuet-1 cultures were grown in LB containing 42 µg/mL chloramphenicol at 37 °C. When the cultures reached OD_600_ ≈ 0.8, protein expression was induced by the addition of 1 mM IPTG and incubated for an additional 3 h. Cells were harvested, lysed and clarified by centrifugation. The supernatant was subjected to IMAC column chromatography followed by Q anion exchange column chromatography to isolate the L-PaF/HT-PcrH complex. HT-PcrH dissociation was achieved by adding 0.1% LDAO and passing the mixture over a final IMAC column with the L-PaF in the flow-through. The protein was dialyzed into storage buffer, pH 6.5 (0.01 M NaH_2_PO_4_/Na_2_HPO_4_, 100 mM NaCl, 5% sucrose, 0.05% LDAO) and stored at −80 °C. Endotoxin levels were verified to be < 5 EU/mg protein using NexGen PTS (EndoSafe cartridges, Charles River Laboratories).

### Preparation of L- PaF BECC438 and BECC470/ME formulations

A squalene-based oil-in-water emulsion was prepared to provide a formulation for this Pa vaccine. Briefly, a homogeneous oil phase was achieved by mixing polysorbate 80 (2% by weight) with squalene (8% by weight). 40 mM histidine (pH 6) and 20% sucrose were added to the oil phase and combined at 7500 rpm using a Silverson L5M-A standard high-speed mixer. This was followed by six passes through a Microfluidics 110P microfluidizer at 20,000 pressure to create a milky emulsion of 4 X ME (MedImmune Emulsion) (32). BECC438b/s (0.5 mg/ml) was made by vortexing it in 0.5% triethylamine, then sonicating it for 30 min at 60 °C in a water bath sonicator until it was fully dissolved. Using 1 M HCl, the BECC438b/s solution pH was adjusted to 7.2. BECC438b/s and ME were combined by vortexing for two min, and then the mixture was incubated overnight at 4 °C. The subsequent day, to get the intended final antigen concentration, L-PaF was combined with the ME-BECC438 solution at a volumetric ratio of 1:1. The BECC470b/s formulation methodology was identical to the BECC438b/s formulation methodology (31, 33).

### Ethics statement

All animal procedures were reviewed and approved by the Institutional Animal Care and Use Committee (IACUC) animal use statement (Protocol 38241) from the University of Missouri.

### Mouse immunization and sample collection

Female C57BL/6J mice aged 6 to 8 weeks (n = 5 per cage) from Jackson Laboratories (Bar Harbor, ME) were used during the study. All groups received vaccinations either intranasally (IN) or intramuscularly (IM), depending on the experimental group’s preferred method. The following formulations were made in 30 µl volumes before dosing. The groups were vaccinated with

1. PBS,
2. 1 μg of L-PaF in ME with 0.5 μg BECC438b (L-PaF/ME/BECC438b).
3. 1 μg of L-PaF in ME with 0.5 μg BECC438s (L-PaF/ME/BECC438s).
4. 1 μg of L-PaF in ME with 0.5 μg BECC470b (L-PaF/ME/BECC 470b).
5. 1 μg of L-PaF in ME with 0.5 μg BECC470s (L-PaF/ME/BECC 470s).

**Figure 1:**
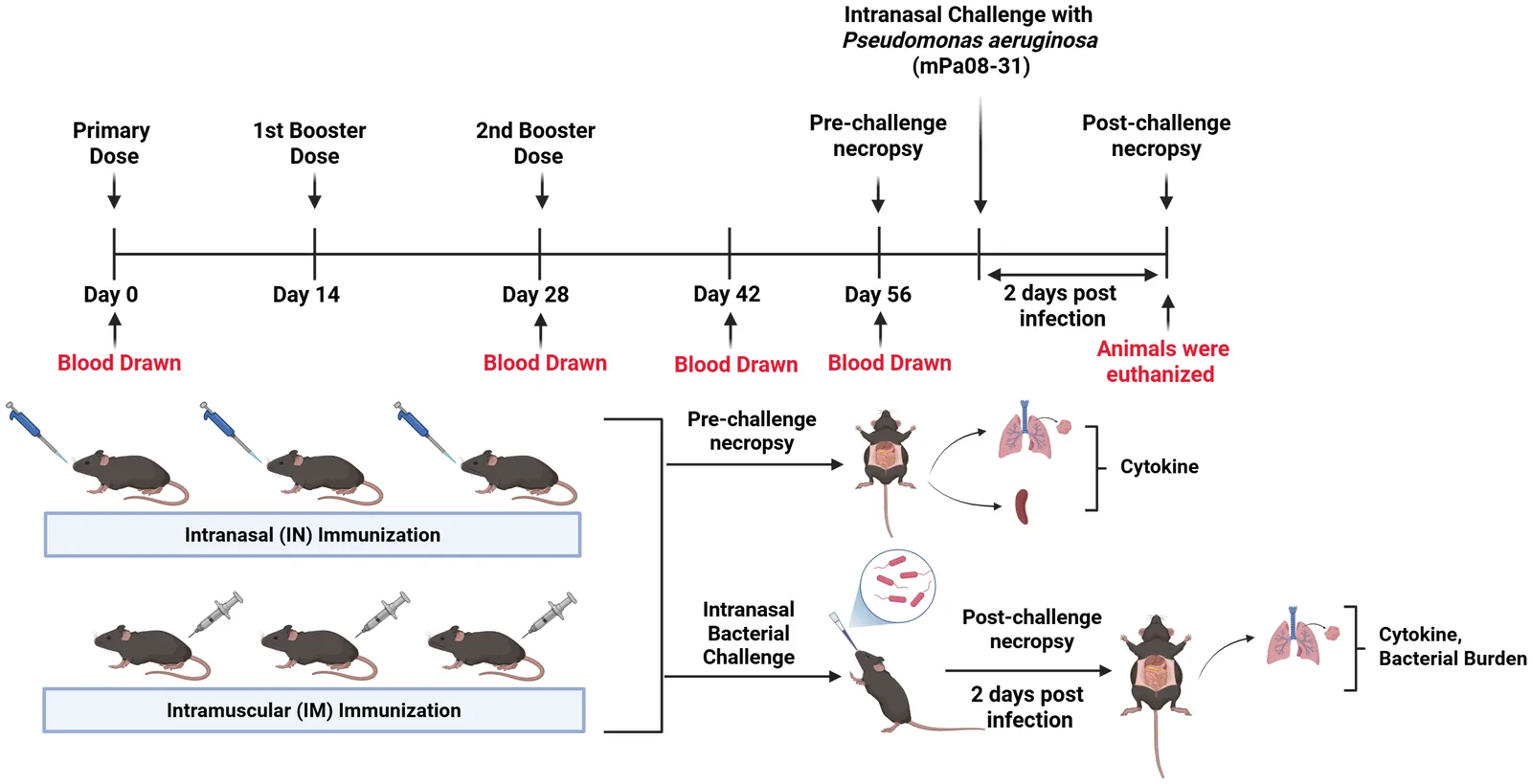
An overview of the experimental setup showing details for each phase of the research. The arrows show the steps in the experimental methods’ advancement and time points. (This figure was generated using BioRender.com).

Mice were anesthetized using isoflurane (~4% in oxygen) dispensed through a precision vaporizer and immunized either IN or IM with the respective formulations. For the IN trials, the indicated formulations were delivered as previously described using a pipette tip to the nares (34). The specified formulations were then made for the IM trials and administered to the inner thigh (33). The initial group size (n = 10 mice/group), the longitudinal blood sampling strategy, with the subsequent division of animals into two terminal sub-cohorts (n = 5 mice/group) for immunological analyses and challenge studies. A summary of the experimental design is described in **Figure 1**. Vaccinations were given on days 0, 14, and 28, and blood samples were taken on days 0, 28, 42, and 56. To evaluate the pre-challenge immunological state, lungs of five mice were removed on day 56. Lastly, five mice were used for the Pa challenge studies. Mice were euthanized using CO_2_ administered at a rate of 30-70% of the chamber volume per minute. Mice were monitored until cessation of respiration and cervical dislocation was performed.

### Enzyme-linked immunosorbent assay

Serum IgG and IgA responses to purified recombinant PcrV and PopB were measured by ELISA as previously described with minor modifications (16). Briefly, 96‑well plates were coated with PcrV or PopB (1 μg/mL in PBS) and blocked overnight at 4 °C with 10% non‑fat dry milk. Serum samples were added and incubated for 1 h at 37 °C. After washing with 0.05% PBS-Tween, anti‑mouse IgG (1:1000, cat. #5450-0011 (474–1806) Sera Care, USA) or IgA (1:4000, cat. #OB1040-05, Southern Biotech, USA) was used as secondary antibodies. Endpoint titers were subsequently measured and expressed as ELISA units per milliliter. IgG1 and IgG3 subclasses were also measured using anti‑mouse IgG1 (1:1000, cat # 1070-05, Southern Biotech, USA) and anti-mouse IgG3 (1:4000, cat # 1191-05, Southern Biotech, USA).

### Opsonophagocytic assay

Opsonophagocytic killing (OPK) assays were performed as described previously, where no exogenous complement source was used (16, 31). Briefly, mPA08‑31 was grown to mid‑log phase and prepared (2 × 10^7^ CFU/mL) in MEM containing 10% BSA. J774A.1 murine macrophages (ATCC, Manassas, VA) were cultured to near (90%) confluence, and the bacterial concentration was adjusted to an MOI of 0.1 in 10% MEM-BSA. Heat‑inactivated sera (at 56 °C for 30 min on day 42 post-immunization) from vaccinated mice were diluted (1:500) and combined with the bacteria and macrophages in 1:1:1 volumetric ratio to reach a final volume of 300 µL, they were then incubated at 37 °C for 30 min. Afterward, samples were serially diluted and plated onto *Pseudomonas* Isolation Agar (PIA) to quantify surviving bacteria. The formula used to measure bactericidal activity was:


$$(CFU\;at\;T_{0})\;-\;(CFU\;at\;T_{30})\;/\;(CFU\;at\;T_{0})\;\times \;100$$

### Bacterial culture and lung challenge

mPA08–31 was incubated overnight at 37 °C with 200 rpm shaking. The culture was diluted 1:100 into fresh low-salt LB medium. mPa08–31 was grown to an A_600_ ~0.3. The bacteria were then adjusted to a concentration of 4 × 10^7^ CFU in 30 µL. IN challenges were performed on 56 days after the initial vaccination under isoflurane anesthesia. Each mouse was given 30 µl of the bacterial suspension. On day 2 post-infection, mice (n = 5) from each group were euthanized, and the lungs were harvested and processed. Lung homogenates were plated on PIA to determine the CFU/lung. Experimental conditions were kept consistent throughout the study (31). The formula used to measure % reduction of lung burden was calculated by:


$$\%\;Reduction\;=\;[\{\frac{PBS-Vaccinated}{PBS}\}\times 100]$$

### Organ processing

The lungs and spleens were aseptically collected in MACS^®^ Tissue Storage Solution (Miltenyi Biotec, USA) and processed using a lung or spleen dissociation kit (Miltenyi Biotec, USA), respectively, into single cell suspensions for immunological investigation. The cell number was adjusted to 1 × 10^7^ cells/ml after an erythrocyte lysis step. Subsequently, these cells were further processed to produce cytokines (see below). Post-challenge necropsies were performed two days after infection, while pre-challenge necropsies were performed 56 days after first vaccination.

### Cytokine determinations

For the pre-challenged cytokine assay, both lung and spleen cells were stimulated for 48 h at 37 °C with 10 μg/ml PcrV, PopB, or PBS. For the post-challenged cytokine assay (*in vivo* pro-inflammatory responses induced by bacterial challenge), lung cells were used without any stimulation. U-PLEX kits were used to analyze the supernatants for the following cytokines: IFN-γ, IL-17A, IL-2, IL-22, IL-6, and TNF-α. An MSD plate reader (Meso Scale Discovery, Rockville, MD) with associated related analytical software (MSD Discovery Workbench) was used to determine the cytokine levels.

### Statistical analysis

Data processing and statistical testing were carried out using GraphPad Prism, version 10.1. To evaluate comparisons between two groups of non-parametric data, the Mann-Whitney test was used. Two-way ANOVA was used to compare three or more groups. PBS served as the control in every instance. *p < 0.05, **p < 0.01, ***p < 0.001, ****p < 0.0001. Unless otherwise indicated in the text or figure legends, studies were carried out with n = 5 mice/group. For correlation analysis, Pearson’s correlation coefficient (r) was calculated and presented with the *p*-value.

## Results

### Evaluation of serum antibody titers against PcrV and PopB induced by BECC438 and BECC470 adjuvants following IN and IM vaccination

**Figure 2:**
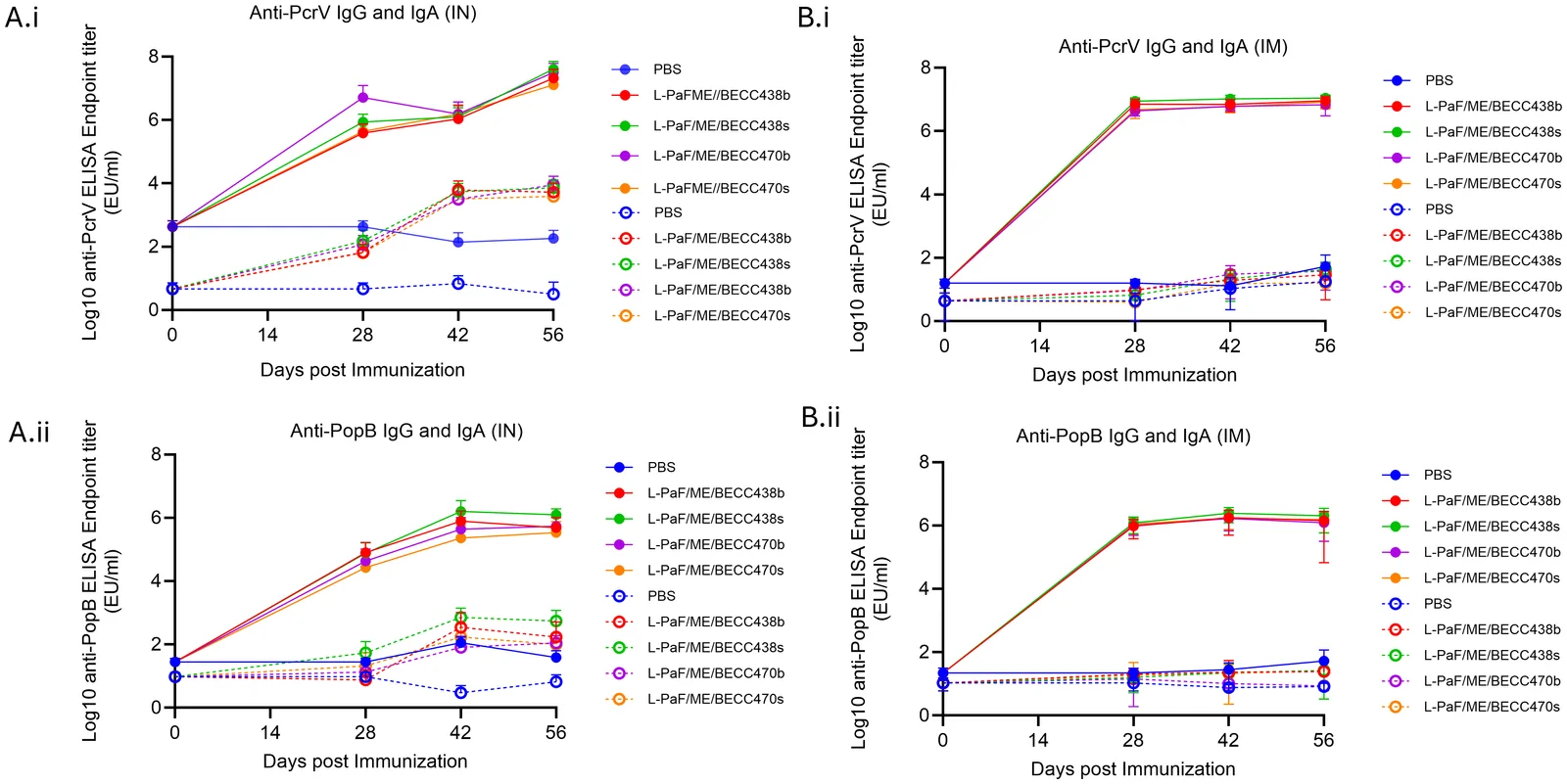
Kinetics of anti-PcrV and anti-PopB serum IgG (closed symbols/solid lines) and IgA (open symbols/dashed lines) titers following vaccination with different BECC adjuvanted L-PaF formulation through intranasal (IN) and intramuscular (IM) route of administration. L-PaF formulations were administered on days 0, 14, and 28. A standard ELISA was used to determine the kinetics of serum antibody titers of different BECC adjuvanted vaccinations, specifically anti-PcrV specific IgG and IgA **(A.i)** for IN administration, **(B.i)** for IM administration) and anti-PopB specific IgG and IgA **(A.ii)** for IN administration, **(B.ii)** for IM administration). IgG titers are represented by solid lines, and the IgA titers are represented by dashed lines. Error bars show the standard deviation (SD) for each group (n = 10 mice/group), whereas each point represents the mean.

**Figure 3:**
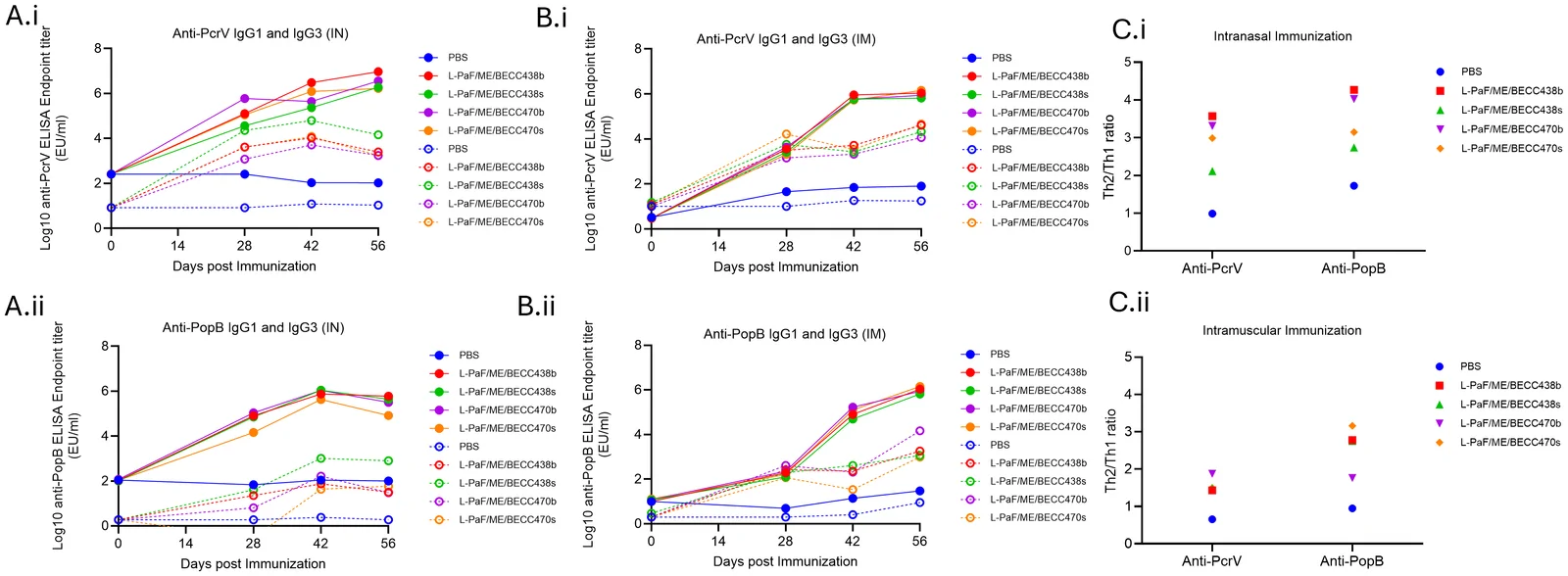
Kinetics of anti-PcrV and anti-PopB serum IgG1 (closed symbols/solid lines) and IgG3 (open symbols/dashed lines) antibody titers following vaccination with different BECC adjuvanted L-PaF formulation through intranasal (IN) and intramuscular (IM) route of administration. Mice were vaccinated on days 0, 14, and 28 with the indicated formulations. A standard ELISA was used to determine the kinetics of serum antibody titers of different BECC adjuvanted vaccinations, specifically of IgG subtypes levels in serum from IN **(A.i, A.ii)**, or IM administration **(B.i, B.ii)** mice for anti-PcrV specific IgG1 and IgG3 **(A.i, B.i)**, and anti-PopB specific IgG1 and IgG3 **(A.ii, B.ii)**. IgG1 titers are represented by solid lines, and the IgG3 titers are represented by dashed lines. Day 56 post immunization sera of IN **(C.i)** and IM **(C.ii)** were used to determine the Th2/Th1 ratio as a measure of Th bias. The unit of measurement for pooled titers was Log10 EU/ml. Each dot in the Th2/Th1 ratio represents a log10 value of [IgG1/IgG3] from EU/ml values of pooled ELISA data. IgG1 and IgG3 was utilized as a complementary Th2 and Th1-associated isotype marker respectively, within our subclass panel to assess relative humoral bias of L-PaF formulation with different adjuvants across different vaccine delivery routes. The IgG1/IgG3 ratio is presented as a descriptive index and not as a statistically tested outcome.

We have previously demonstrated that IN in mice with L-PaF formulated with ME and BECC438b elicits a protective immune response against both PcrV and PopB (31). To determine any potential differences in the effect of BECC438 or BECC470 with L-PaF/ME administered by the IN and IM routes, mice were vaccinated using a prime-boost-boost scheme with 14-day intervals (see **Figure 1**). We have evaluated the complete antibody profile induced by different BECC adjuvanted L-PaF/ME formulations following IN immunization route. L-PaF/ME formulations containing BECC438 or BECC470 robustly induced PcrV- and PopB-specific serum IgG antibodies (**Figures 2A.i, A.ii**), with comparable titers reached by day 42 in most cases and by day 56 in all cases. IN vaccination also resulted in much higher PcrV-specific and PopB-specific IgA titers than PBS vaccinated group (**Figures 2A.i, A.ii**). IM immunization with all adjuvanted formulations successfully induced PcrV- and PopB-specific serum IgG, with comparable titers reached by day 28, 42 and 56 in all cases (**Figures 2B.i, B.ii**). However, IM immunization failed to generate higher PcrV- and PopB-specific IgA titers than PBS vaccinated group (**Figures 2B.i, B.ii**). Previous studies have shown that elevated IgG1 levels correlate with enhanced opsonophagocytic activity, whereas IgG3 is more effective at complement fixation (35). When analyzing IgG subclasses, compared to the sera from PBS vaccinated mice, all L-PaF/ME formulated BECC adjuvanted groups possessed higher PcrV- and PopB-specific titers for IgG1 and IgG3 subclasses, however differences were comparable between two adjuvants (**Figures 3A.i , A.ii**) with IgG1 acting as the predominant isotype. At the subclass level IM immunization also drives high PcrV- and PopB-specific IgG1 and IgG3 levels compared to PBS controls (**Figures 3B.i, B.ii**). Balanced Th2/Th1 ratio against both PcrV and PopB following IN immunization was most consistently achieved by the L-PaF/ME/BECC438 formulations suggesting coordinated humoral and cellular immune activation, potentially driven by mucosal immune engagement (**Figure 3C.i**). However, the resulting Th2/Th1 ratios following IM immunization revealed a distinct systemic bias, characterized by a marginal Th1 skew against PcrV and a Th2 skew against PopB (**Figure 3C.ii**). Th1-skewed responses were generated by IM vaccination as reflected by higher IgG3 levels. No substantial differences were observed between biologically derived and synthetic BECC438 or BECC470 formulations under IN or IM immunization.

### Opsonophagocytic activity of serum immunoglobulins with L-PaF/ME formulated BECC438 or BECC470 adjuvants following IN and IM vaccination

**Figure 4:**
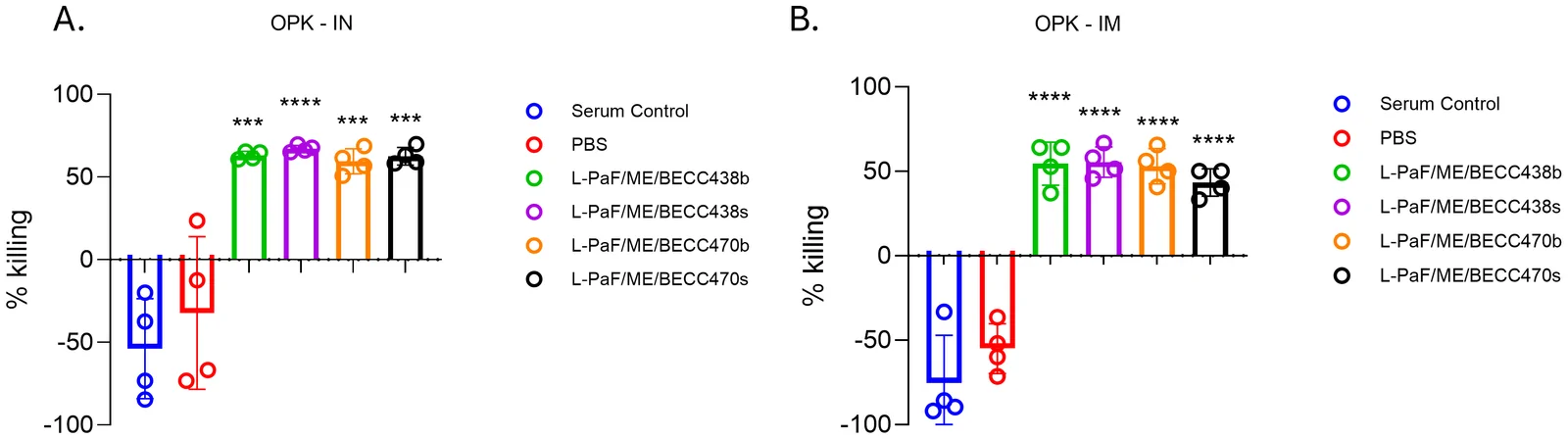
BECC438s promotes optimal opsonophagocytic killing (OPK) activity for vaccinated mouse serum immunoglobulins. Day 42 sera from intranasal **(A)** and intramuscular **(B)** vaccinated groups were heat-treated at 56 °C for 30 min for subsequent use in OPK assays. Briefly, the murine macrophage cell line J774A.1 was combined with clinical Pa strain mPa08–31 at an MOI of 0.1. Heat-inactivated serum was mixed at a ratio of 1:500. OPK activity was determined at 30 min using the following formula: [{(T0-T30)/T0}x100] where T0 and T30 were CFUs at time 0 and 30min, respectively. OPK activities are shown for each vaccinated group with PBS and non-immune serum controls. Statistical analysis was performed by two-way ANOVA (Dunnett’s multiple comparisons test). Error bars represent SD, and each experiment was performed at least twice with quadruple technical repeats, ***p < 0.001, ****p < 0.0001. Exact p values can be found in **Supplementary Tables 1**, **2**.

To assess the functional activity of vaccine-induced serum antibodies, *in vitro* opsonophagocytic killing (OPK) assays were performed. In contrast to binding assays such as ELISA, OPK assays capture the biological relevance of antibodies as opsonins by measuring their capacity to engage Fc receptors and drive phagocytosis and intracellular killing. OPK activity (**Figure 4**) was markedly higher in all L-PaF/ME BECC formulated groups compared to the PBS vaccinated mice, which exhibited negative killing (an indicator of bacterial growth over the course of the assay). Negative percent killing values in the PBS vaccinated group and non-immune serum controls underscore the inability of phagocytes to efficiently clear Pa in the absence of opsonic antibodies. We evaluated the functional antibody profiles generated by the IN immunization route (**Figure 4A**). Within the IN vaccinated group, distinct differences in adjuvant performance were observed; L-PaF/ME/BECC438s produced the highest level, reaching approximately 70% killing. The next most active formulation included BECC438b, with activity comparable to BECC470s (**Figure 4A**). IM immunization with all BECC adjuvanted L-PaF/ME formulation resulted in only ~50% OPK activity (**Figure 4B**). OPK activity has been proposed as a key correlate of protection for Pa vaccines (36), consistent with findings from pneumococcal and meningococcal vaccine studies where opsonic function serves as a surrogate marker of protective immunity (37, 38). The sera were heat-inactivated prior to the assay and no exogenous complement source was included. The observed bactericidal activity primarily reflects Fc receptor-mediated opsonophagocytic uptake and killing by macrophages rather than complement-dependent bacterial killing. Therefore, the contribution of vaccine-induced serum antibodies should be interpreted in the context of antibody-mediated opsonization and macrophage effector function rather than complement activation.

### Cellular immune profiles elicited by BECC-adjuvanted formulations following IN and IM vaccination

**Figure 5:**
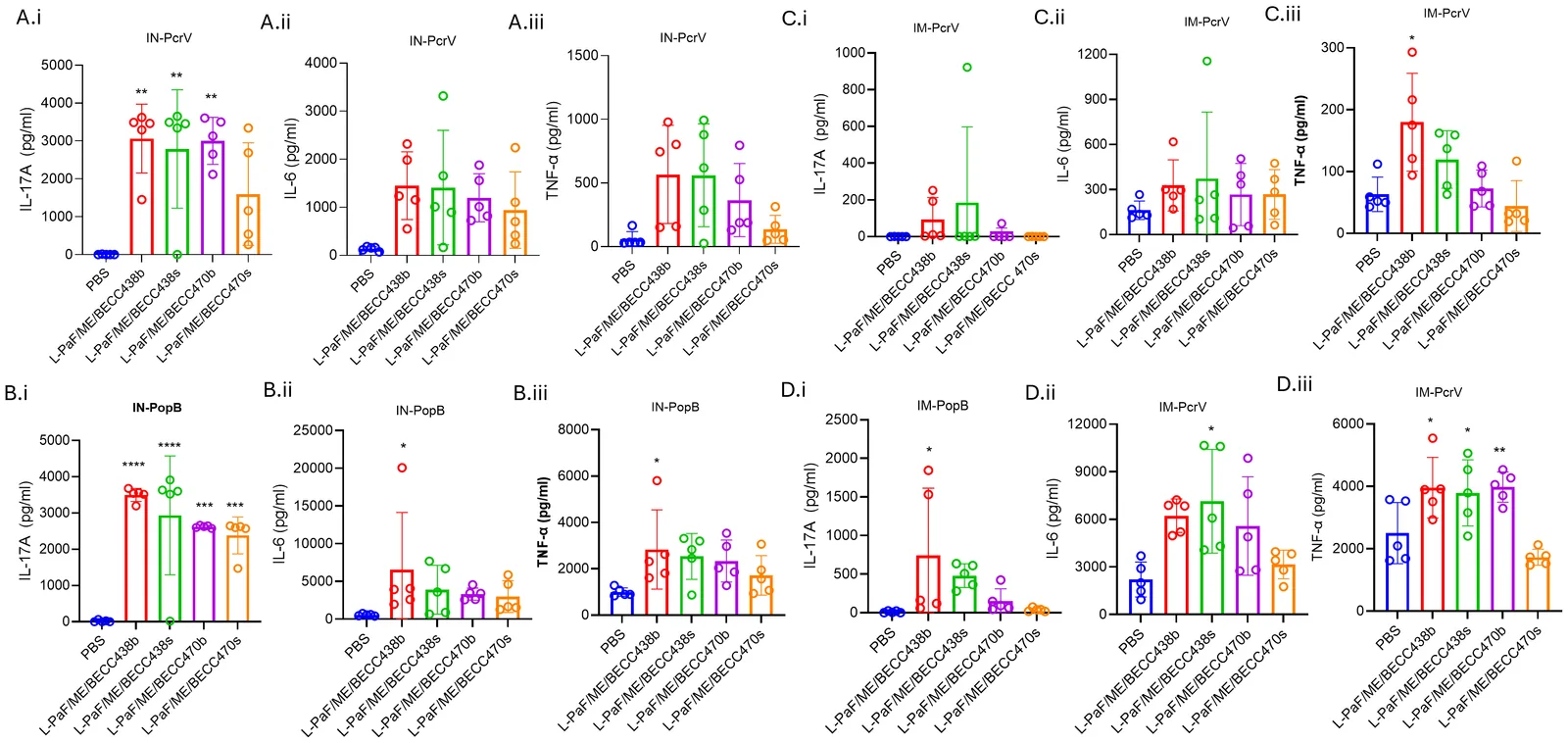
Quantification of pro-inflammatory cytokines secreted from lung cells after stimulation with PcrV and PopB. Cytokine secretion from lung cells from intranasal (IN) immunized **(A.i-iii, B.i-iii)** and intramuscular (IM) immunized **(C.i-iii, D.i-iii)** pre-challenged mice following stimulation with PcrV **(A.i-iii, C.i-iii)** or PopB **(B.i-iii, D.i-iii)** is presented. Single cell suspensions of lung cell were treated with 10 μg/ml of PcrV or PopB protein and incubated for 48 h at 37 °C. Secretion of IL-17A **(A.i, B.i, C.i, D.i)**, IL-6 **(A.ii, B.ii, C.ii, D.ii)**, and TNF-α **(A.iii, B.iii, C.iii, D.iii)** was determined by Meso Scale Discovery (MSD) analysis as per the manufacturer’s instructions and was presented as pg/ml/10^6^ cells. Data are plotted as actual values from individuals ± SD (n=5) in each group. Statistical significance was calculated by comparing the PBS group with their immunized counterparts using two-way ANOVA (Dunnett’s multiple comparisons test), *p < 0.05, **p < 0.01, ***p < 0.001, ****p < 0.0001. Exact p-values can be found in **Supplementary Table 3**.

**Figure 6:**
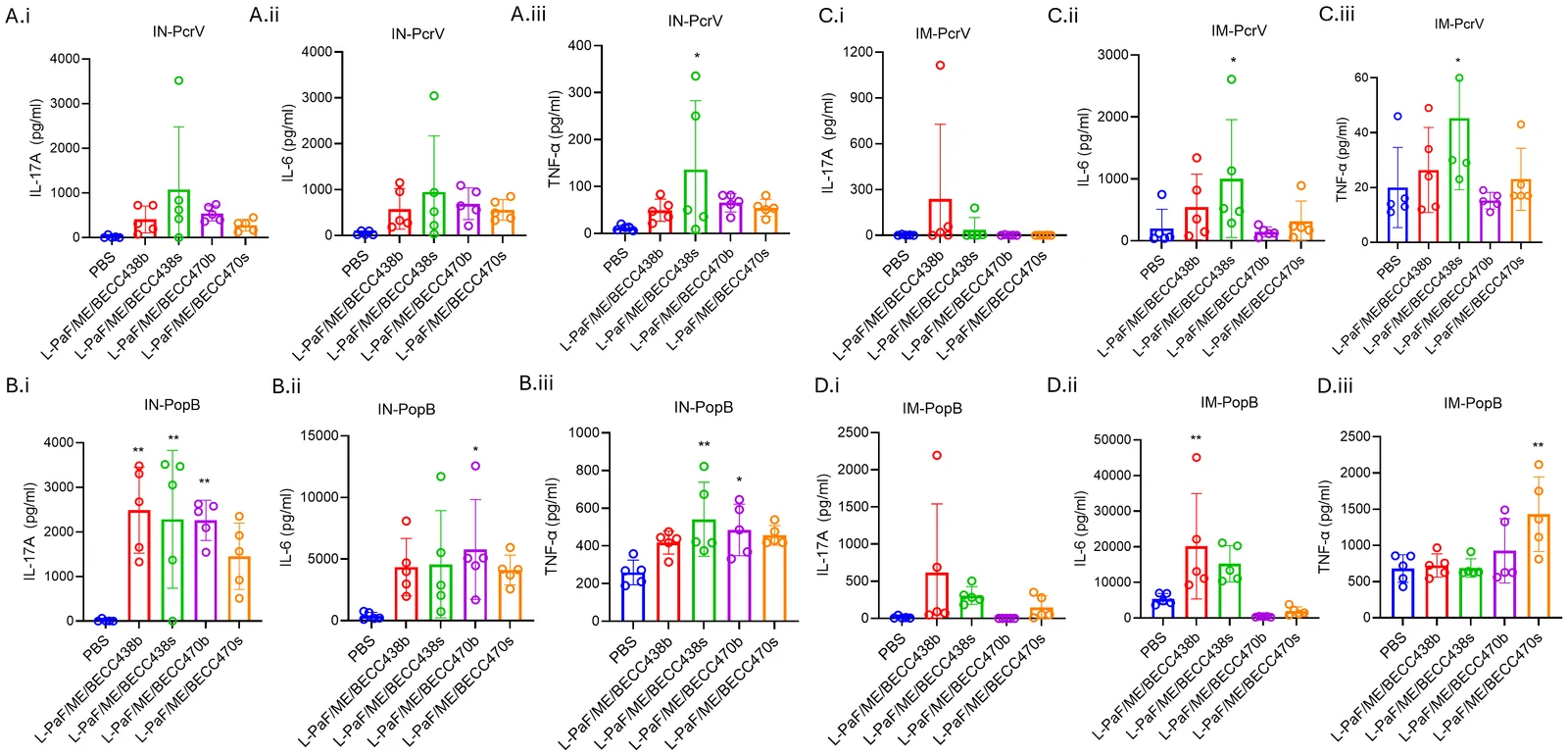
Quantification of pro-inflammatory cytokines secreted from splenocytes after stimulation with PcrV and PopB. Cytokines secreted from splenocytes from intranasal (IN) immunized **(A.i-iii, B.i-iii)** and intramuscular (IM) immunized **(C.i-iii, D.i-iii)** pre-challenged mice following stimulation with PcrV **(A.i-iii, C.i-iii)** or PopB **(B.i-iii, D.i-iii)** are presented. Splenocytes were treated with 10 μg/ml of PcrV or PopB protein and incubated for 48 h at 37 °C. Secretion of IL-17A **(A.i, B.i, C.i, D.i)**, IL-6 **(A.ii, B.ii, C.ii, D.ii)**, and TNF-α **(A.iii, B.iii, C.iii, D.iii)** was determined by Meso Scale Discovery (MSD) analysis as per the manufacturer’s instructions and was presented as pg/ml/10^6^ cells. Data were plotted as actual values from individuals ± SD (n=5) in each group. Statistical significance was calculated by comparing the PBS group with their immunized counterparts using two-way ANOVA (Dunnett’s multiple comparisons test), *p < 0.05, **p < 0.01. Exact p-values can be found in **Supplementary Table 5**.

To define the cellular immune mechanisms elicited by vaccine adjuvant compositions and route of administration and their potential contribution to protective immunity, lung cells and splenocytes were harvested from mice, 56 days after the first vaccination. Cytokine secretion was assessed after stimulating these cells with PcrV or PopB. We evaluated the immune profiles generated by the IN route (**Figures 5A.i-iii, B.i-iii**). Lung cells from mice vaccinated IN with L-PaF/ME/BECC438b and L-PaF/ME/BECC438s groups produced significantly more IL-17A (**Figures 5A.i, B.i**) and IL-2 (**Supplementary Figures 1A.ii, B.ii**) after PcrV and PopB stimulation than PBS vaccinated groups, while the L-PaF/ME/BECC438b group showed significantly more IL-6, TNF-α, and IFN-γ (**Figures 5B.ii, B.iii**; **Supplementary Figure 1B.i**). L-PaF/ME/BECC438s induced a significant increase in IL-22 following PopB stimulation (**Supplementary Figure 1B.iii**). Similarly, L-PaF/ME/BECC470b elicited significantly elevated IL-17A responses after PcrV and PopB stimulation (**Figures 5A.i, B.i**, respectively), along with increased IL-2 production following PcrV stimulation (**Supplementary Figure 1A.ii**). In addition, IN immunization with L-PaF/ME/BECC470s resulted in significantly higher IL-17A levels after PopB stimulation compared to PBS-vaccinated controls (**Figure 5A.ii**). Additionally, splenocytes from mice vaccinated IN with L-PaF/ME/BECC438s and L-PaF/ME/BECC438b groups showed significant IL-17A induction (**Figure 6B.i**) after PopB stimulation. Splenocytes from the L-PaF/ME/BECC438s vaccinated group also showed TNF-α induction (**Figures 6A.iii, B.iii**) after PcrV and PopB stimulation, respectively. The L-PaF/ME/BECC470b vaccinated group maintained significant induction of IL-17A, IL-6, TNF-α, IFN-γ (**Figures 6B.i, B.ii, B.iii**; **Supplementary Figure 2B.i**) after PopB stimulation and IL-2 (**Supplementary Figure 2A.ii**) after PcrV stimulation. Meanwhile, the L-PaF/ME/BECC470s vaccinated group only showed significant IFN-γ (**Supplementary Figure 2B.i**) induction after PopB stimulation.

In contrast to the above, IM immunized L-PaF/ME/BECC438b mouse lung cells showed significant IL-17A and IFN-γ induction (**Figure 5D.i**; **Supplementary Figure 1D.i**) after PopB stimulation and TNF-α induction (**Figures 5C.iii, D.iii**) in response to PcrV and PopB. IM immunized L-PaF/ME/BECC438s mouse lung cells showed significant IL-6, TNF-α, IFN-γ, and IL-2 induction (**Figures 5D.ii, D.iii**; **Supplementary Figures 1D.i, D.ii**), but only after PopB stimulation. In the L-PaF/ME/BECC470b group vaccination mice, the lung cells also showed induction of TNF-α (**Figure 5D.iii**) after PopB stimulation. The IM vaccinated L-PaF/ME/BECC438b mouse splenocytes had induce IL-6, IFN-γ, IL-2, and IL-22 induction (**Figure 6D.ii**; **Supplementary Figures 2D.i, D.ii, D.iii**) and the L-PaF/ME/BECC438s vaccinated group showed induction of IFN-γ, IL-2, IL-22 (**Supplementary Figures 2D.i, D.ii, D.iii**) after PopB stimulation and IL-6, TNF-α (**Figures 6C.ii, C.iii**) after PcrV stimulation in these *ex vivo* studies. The L-PaF/ME/BECC470s vaccinated mouse splenocytes also showed significant induction of TNF-α and IL-2 (**Figure 6D.iii**; **Supplementary Figure 2D.ii**) while the L-PaF/ME/BECC470b had significantly lower IFN-γ (**Supplementary Figure 2D.i**) after PopB stimulation.IM vaccination appears to show a systemic Th1-skewed profile of cytokine induction with less mucosal imprinting when compared to the IN vaccinations. These distinct cytokine patterns underscore the fundamentally different immunological programming associated with each route.

### L-PaF/ME formulated BECC438s adjuvant enhances bacterial clearance from the lungs of IN vaccinated mice more effectively than IM delivery

**Figure 7:**
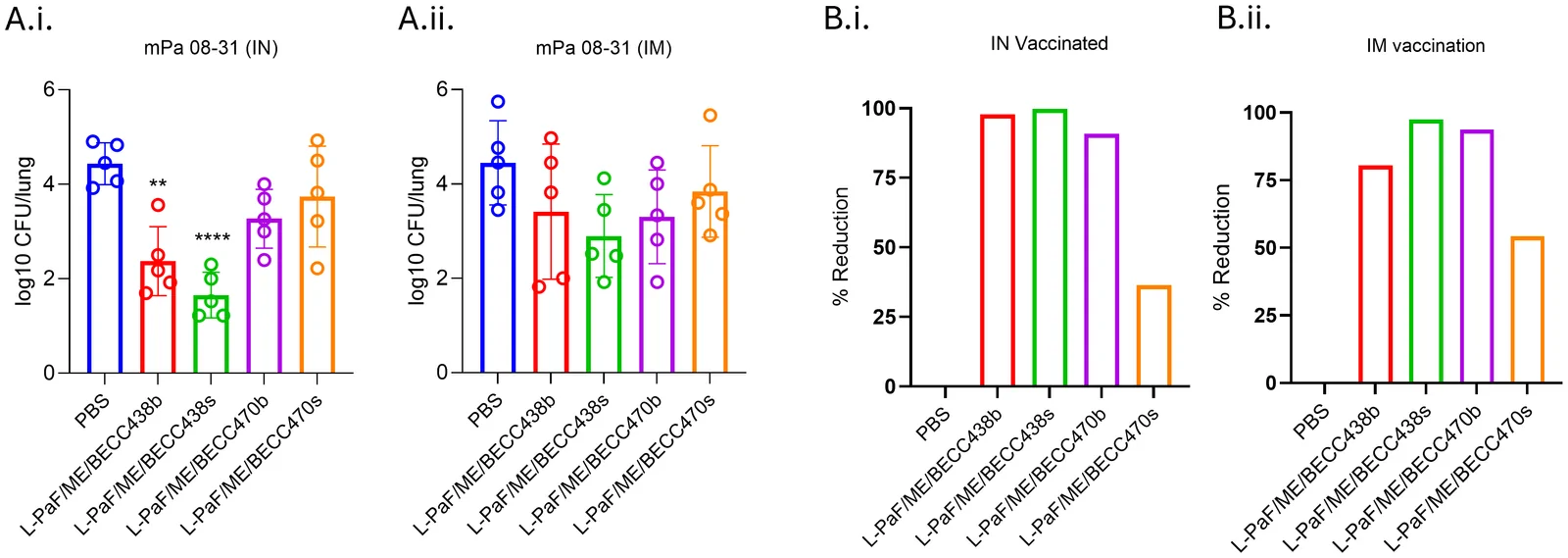
Intranasal vaccination with BECC438s promotes increased bacterial clearance from the lungs of vaccinated mice. On day 56 post immunization, mice were challenged IN with clinical Pa strain mPa08–31 at a concentration of 4x10^7^ CFU/30µl/mouse, and CFU/lung were determined on 2 DPI. **(A.i.)** The Pa lung burdens of IN immunized L-PaF formulation groups were compared to the PBS group and **(A.ii.)** The Pa lung burdens of IM immunized L-PaF formulation groups were compared to the PBS group represented in the graph (n = 5). The points represent individual CFU/lung values, and the SD are denoted by error bars. **(B.i.)** & **(B.ii.)** Bacterial reduction percentage (%) of lung burden due to IN vaccination & IM vaccination of different L-PaF formulations group respectively. % Reduction activity was determined using the following formula: [{(PBS − Vaccinated)/PBS} ×100]. Lung burden was compared between PBS and all the other groups using statistical analysis by two-way ANOVA (Dunnett’s multiple comparisons test), **p < 0.01, ****p < 0.0001. Exact p-values can be found in **Supplementary Tables 7**, **8**.

To evaluate the protective efficacy of the vaccine formulations, mice were challenged with mPa08-31, and the lung Pa burden quantified (**Figure 7**). Within the IN immunized group, the BECC438 variants demonstrated superior protective efficacy. Mice immunized with L-PaF/ME/BECC438s exhibited significantly lower (p<0.0001) lung bacterial burden compared to the PBS vaccinated group (**Figure 7A.i**). The L-PaF/ME/BECC438b vaccinated mice also had significantly lower (p<0.01) lung bacterial burden compared to the PBS vaccinated group (**Figure 7A.i**), though to a lesser degree. The overall percent reduction of Pa burden in the lungs of IN immunized L-PaF/ME/BECC438s group was consistently high (≥99% reduction), followed by L-PaF/ME/BECC438b (≥97% reduction), as compared to the PBS group. Mice vaccinated IN with L-PaF/ME/BECC470b showed an ~90% reduction, whereas L-PaF/ME/BECC470s showed an ~36% reduction of Pa lung burden (**Figure 7B.i**; **Supplementary Tables 8**). IM immunization did not result in a statistically significant reduction in lung burden in L-PaF groups compared to PBS (**Figure 7A.ii**), and this was also true of the mice vaccinated with L-PaF/ME formulated with either BECC470s or BECC470b (**Figure 7Ai**). The IM immunized L-PaF/ME BECC-adjuvanted formulations resulted in a reduction up to about 80% for BECC438b, ~97% for BECC438s, ~93% for BECC470b, and 54% for BECC470s as the adjuvants used (**Figure 7B.ii**; **Supplementary Tables 8**).

The *in vivo* protection presented in **Figure 7** positively correlates with *in vitro* OPK activity (**Figure 4A**) as described previously for IN vaccination (39). IM vaccinated L-PaF formulation groups failed to significantly lower the number of Pa to give *in vivo* protection (**Figure 7A.ii**) to mucosal (IN) challenge, though they do have a relatively high level of bacterial burden reduction *in vivo* (**Figure 7B.ii**) and significant *in vitro* bacterial killing activity (**Figure 4B**) and presence of IgG but with a low IgA titer (**Figures 2B.i, B.ii**). These findings underscore the importance of mucosal delivery (IN) in inducing localized respiratory immunity, including IgA responses, which are not consistently achieved with systemic (IM) immunization (40, 41).

### Evaluation of BECC-adjuvanted vaccine formulations identifies route-dependent cytokine dynamics as key drivers of mucosal protection

**Figure 8:**
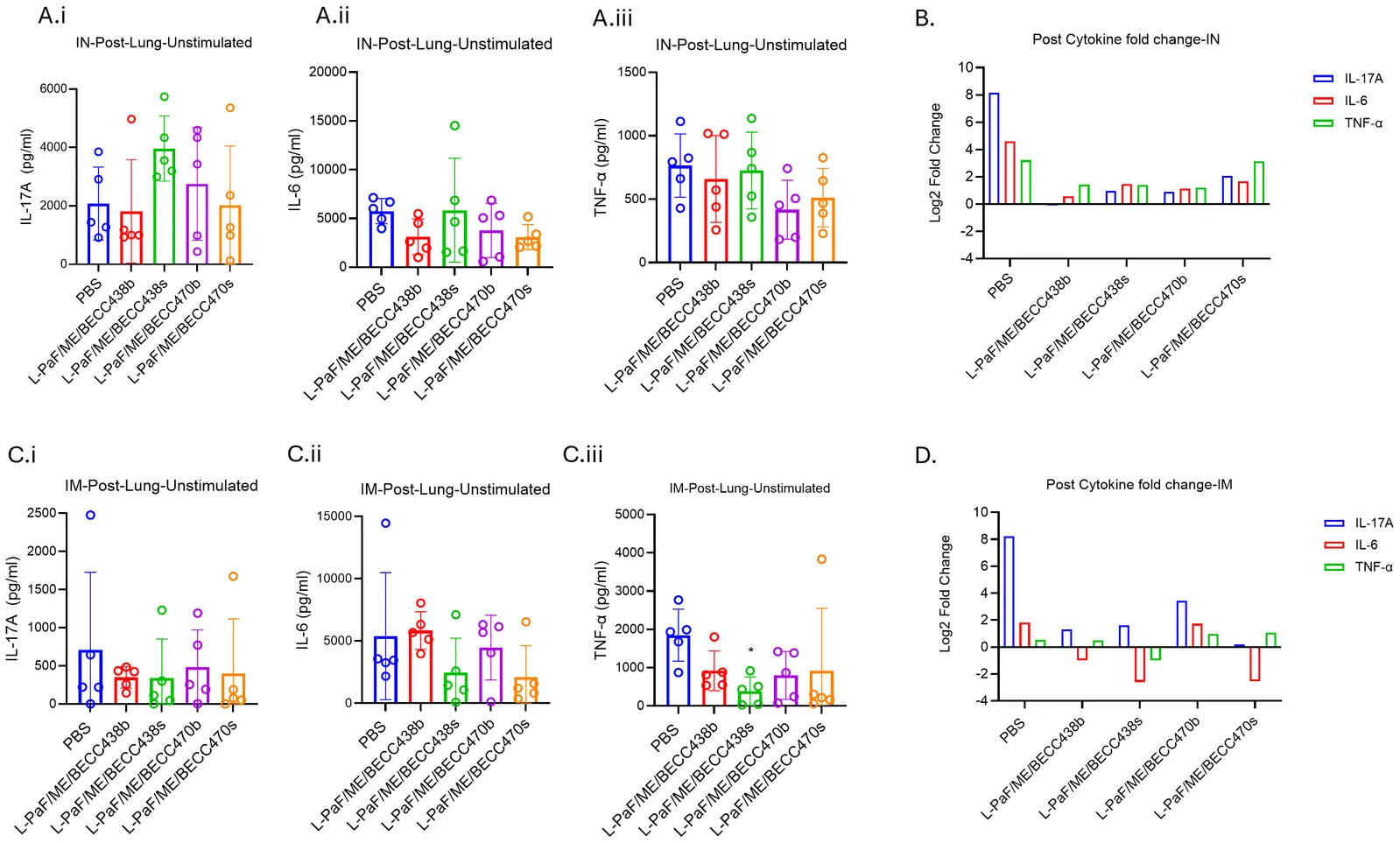
Post-challenge cytokine responses and fold change of cytokine to pre-challenge unstimulated lung cells. Data for lung cells from intranasal (IN) immunized **(A.i-iii)** and intramuscular (IM) immunized **(B.i-iii)** post challenged (with the Pa isolate mPa08-31) mice are presented. Lung cell suspensions were left untreated for 48 h at 37 °C. IL-17A **(A.i**, **B.i)**, IL-6 **(A.ii**, **B.ii)** and TNF-α **(A.iii**, **B.iii)** were measured by Meso Scale Discovery (MSD) analysis as per manufacturer’s instructions and were presented as pg/ml/106 cells and plotted as actual values from individuals ± SD (n=5) in each group. log_2_ fold changes between pre-challenge and post-challenge unstimulated lung cytokines were evaluated for IN immunization **(C)** and IM immunization **(D)**. Statistical significance was analysis by comparing the PBS group with and all the other groups using two-way ANOVA (Dunnett’s multiple comparisons test), *p < 0.05, **p < 0.01, ***p < 0.001. Exact p values can be found in **Supplementary Tables 9**, **11**.

**Figure 9:**
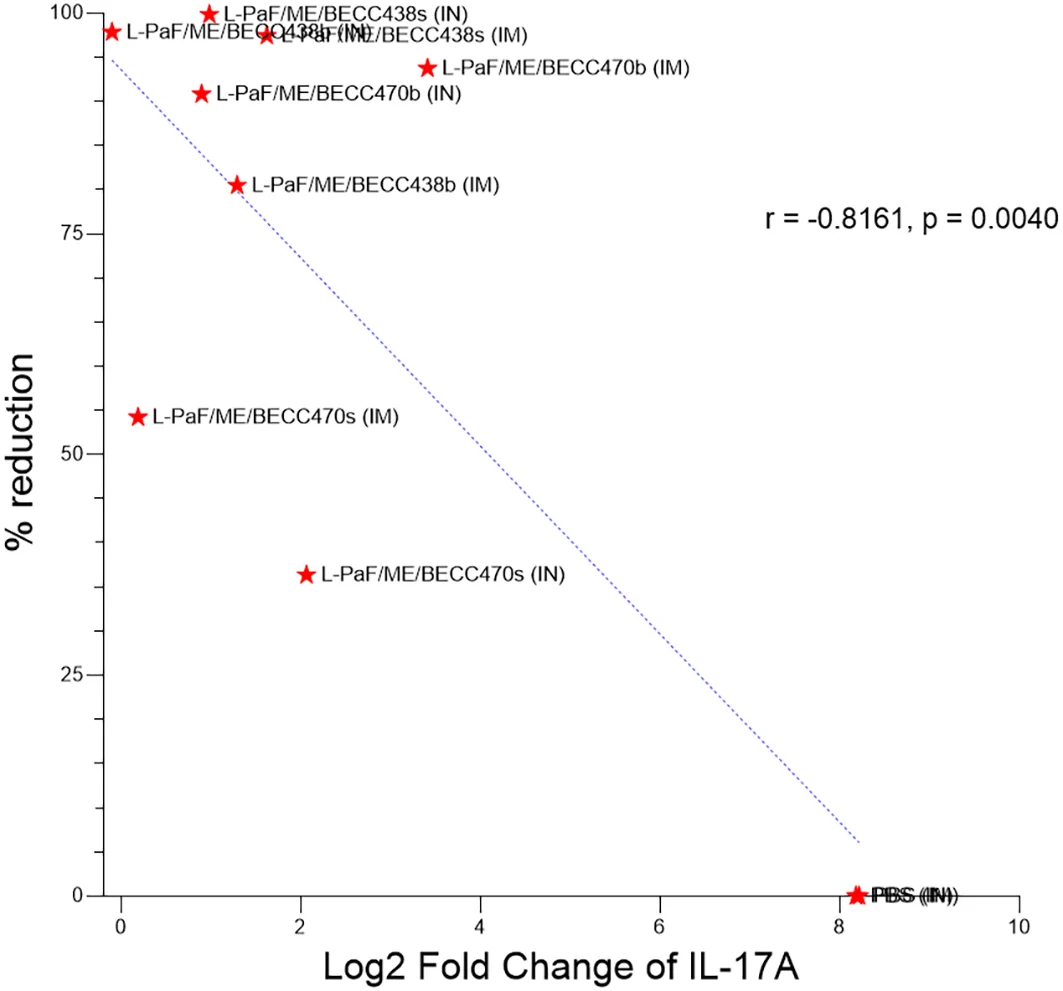
Scatter plot showing the correlation between Log2 Fold change of IL-17A (Pre- & Post-infection unstimulated ex-vivo lung cells secreted IL-17A) and in-vivo % reduction of bacterial burden in lung. The X-axis shows values of Log_2_ fold change of IL-17A, and the Y-axis shows % reduction of bacterial burden in the lung. Pearson’s r coefficient and simple linear regression (95% confidence level) were calculated for the correlation study. The dashed line represents the linear regression fit (r = -0.8161, p = 0.0040). A negative correlation was observed between % reduction and Log_2_ fold change of IL-17A. Exact p-values can be found in **Supplementary Table 12**.

A comparison of post-challenge cytokine profiles reveals key differences associated with protective outcomes. To define these infection-driven responses, lung cells were collected from mice on day 2 post-infection and evaluated without *ex vivo* stimulation (**Figure 8**). Because these cells were not stimulated with purified antigens, these measurements reflect the immediate host response to acute infection rather than antigen-specific recall. Among the L-PaF/ME with BECC-adjuvanted formulation groups of IN or IM vaccinated, do not appear to show any significant cytokine upregulation (associated with a more regulated pro-inflammatory response) relative to the PBS vaccinated group (**Figures 8A.i-iii, B.i-iii**). The single exception is for downregulation of TNF-α in the L-PaF/ME/BECC438s vaccinated group following IM immunization (**Figure 8B.iii**). We also tested pre-challenged, unstimulated lung cells and found that L-PaF/ME/BECC438b and L-PaF/ME/BECC438s groups of IN immunized have significant induction of IL-17A, IL-6 (**Supplementary Figure 3A.i-ii**) compared to the PBS vaccinated group. L-PaF/ME/BECC438b or L-PaF/ME/BECC438s, of IM vaccinated mice had significantly increased IL-6 (**Supplementary Figure 3B.ii**), whereas TNF-α was significantly reduced (**Supplementary Figure 3B.iii**). A similar pattern was observed in the L-PaF/ME/BECC470b and L-PaF/ME/BECC470s IM vaccinated groups’ pre-challenged lung cells had a significant reduction of TNF-α (**Supplementary Figure 3B.iii**), and the L-PaF/ME/BECC470s group had induction of IL-6 (**Supplementary Figure 3B.ii**) even without any stimulation. Whereas IL-17A levels were comparable between L-PaF/ME BECC-adjuvanted vaccinated and PBS control groups following IM immunization (**Supplementary Figure 3B.i**). However, analyzing the log_2_ fold-change from pre-challenge baseline to post-challenge infection revealed distinct adjuvant-dependent patterns (**Figures 8C, D**), while PBS-vaccinated mice experienced a massive, dysregulated inflammatory surge. When evaluating the log_2_ fold-change post-challenge, the PBS vaccinated groups responded to the mPa08–31 challenge by exhibiting a considerable rise in pro-inflammatory cytokines (IL-17A ≥ 8 log_2_, IL-6 ≥ 4 log_2_, TNF-α ≥ 3 log_2_ fold change in IN sets and IL-17A ≥ 8 log_2_, IL-6 ≥ 1.8 log_2_, TNF-α ≥ 0.5 log_2_ fold change in IM sets) (**Supplementary Tables 11**). Following IN administration, all L-PaF/ME BECC formulation vaccinated groups exhibited an approximate 2 log_2_ fold increase in these cytokines, whereas, with the exception of the L-PaF/ME/BECC470b group patterns (**Figure 8C**). In IM immunization resulted in reduced IL-6 and TNF-α levels post-challenge relative to pre-challenge baseline patterns (**Figure 8D**). All groups immunized with the L-PaF/ME BECC formulations displayed a noticeable decrease in these cytokines after infection (**Figures 8C, D**; **Supplementary Tables 11**) (33, 42). To determine how this different adjuvant formulation with route-specific inflammatory dynamics influenced protection, we correlated the log_2_ fold-change of IL-17A (pre- vs. post-challenge unstimulated cells) against the absolute reduction of *in vivo* bacterial burden across both delivery systems. A negative correlation (r = -0.8161, p = 0.0040) was observed between log_2_ fold change of IL-17A (pre- vs post-challenge unstimulated cells) and the reduction of *in vivo* bacterial burden after IN or IM immunization (**Figure 9**). In summary, vaccinated groups demonstrated a controlled cytokine response following the live bacterial challenge. In contrast, the PBS vaccinated groups exhibited a more vigorous response, which was associated with significantly higher morbidity and a greater bacterial burden.

## Discussion

The development of an effective vaccine against Pa remains a challenge due to its intrinsic resistance mechanisms, antigenic variability, and ability to persist in host tissues. These attributes render most conventional therapeutic strategies ineffective. Pa is an opportunistic pathogen and a leading cause of chronic and acute respiratory infections in people with cystic fibrosis (CF), chronic obstructive pulmonary disease (COPD), and those requiring mechanical ventilation. Despite decades of research, no licensed vaccine currently exists against Pa, underscoring the urgency of developing novel immunization strategies that can overcome these barriers. Our previous work demonstrated that formulation of L-PaF with ME and BECC438b elicits a balanced Th1/Th2 immune response when delivered IN and confers enhanced protection against Pa challenge (31). The route of vaccine administration can play a pivotal role in shaping the quality, localization, and durability of the immune response. Building on this, we evaluated the immunogenicity and protective efficacy of L-PaF/ME formulated with a reduced dose (0.5 µg) of different BECC forms, BECC438b, BECC438s, BECC470b, and BECC470s, administered through either IN or IM routes.

All formulations induced robust IgG responses regardless of the route of administration; however, IgA levels were markedly lower following IM immunization, underscoring the superior capacity of the IN route to engage in mucosal immunity. The detection of serum IgG against PcrV and PopB of Pa suggests the activation of a systemic humoral response in the vaccinated mice. Meanwhile, the presence of IgA detected in serum samples against these antigens reflects a parallel activation of the mucosal immune response. A successful vaccine against Pa should activate both mucosal and systemic immunity, eliciting strong IgG and IgA responses that enhance bacterial clearance against opportunistic mucosal pathogens such as Pa (43, 44). Several experimental vaccine platforms targeting Pa, have demonstrated the induction of systemic humoral immunity, including both IgG and IgA responses, in murine models. Study showed that IN immunization in mice with a self-adjuvanting fusion protein composed of PcrV, PopB, and the A1 subunit of dmLT led to strong serum IgG and IgA titers, enhanced IL-17A expression, and accelerated bacterial clearance from the lungs (16). Another study showed that mice immunized with a hybrid protein composed of Exotoxin S and PcrV formulated with alum or MPL adjuvants exhibited significantly elevated levels of serum IgG and IgA, alongside mucosal IgG and IL-17A, indicating a multifactorial immune response capable of reducing bacterial burden in the urinary tract (44). Earlier studies using recombinant outer membrane proteins OprF and OprI demonstrated induction of high serum IgG titers and protective efficacy in murine pneumonia models. While mucosal IgA was less pronounced, systemic IgA responses were detectable and correlated with reduced bacterial burden (45).

In this study, the highest opsonophagocytic killing activity was observed following immunization via the IN route in comparison to IM administration. OPK reflects the biological relevance of antibodies by assessing their ability to engage Fc receptors and complement pathways, thereby promoting phagocytosis and intracellular killing (46–48). This trend aligns with mucosal immunization’s propensity to induce both systemic and local IgG/IgA responses, which may enhance functional antibody quality through affinity maturation and isotype diversification (41). Comparatively, IM immunization primarily elicits systemic IgG, which, while effective in OPK, may lack the mucosal imprinting that optimizes opsonic function against respiratory pathogens (49). Among all groups, the L-PaF/ME/BECC438s formulation consistently demonstrated the highest *in vivo* protective efficiency, regardless of the route of administration. This suggests that structural or compositional differences between the bis-phosphorylated BECC438 and mono-phosphorylated BECC470 adjuvant systems may influence antibody avidity or Fc‑mediated effector functions slightly differently, a phenomenon also observed in other vaccine platforms where adjuvant chemistry modulates IgG subclass distribution. Also, this superior performance of BECC438s suggests that differences between the biologically derived (BECC438b) and chemically synthesized BECC438 (BECC438s) formulations may influence the quality of the resulting immune response. Unlike biologically derived BECC438b, which is produced through bacterial biosynthesis and contains minor lipid A congeners resulting from natural enzymatic variability during fermentation, BECC438s is generated through total chemical synthesis and possesses a highly defined and homogeneous molecular structure (27). Greater structural uniformity may improve the consistency of interactions with the MD-2/TLR4 receptor complex, reducing the presence of lipid species that could exhibit altered agonist activity or compete for receptor engagement (27). Although the present study was not designed to directly evaluate receptor-binding kinetics or formulation biophysics, the enhanced functional antibody responses and superior protection observed with BECC438s are consistent with the hypothesis that a chemically defined adjuvant can provide more predictable innate immune activation. In addition, the high purity and batch-to-batch reproducibility achievable through chemical synthesis may reduce variability in downstream immune responses and facilitate manufacturing, quality control, and regulatory assessment (27). These attributes are particularly attractive for clinical translation, where maintaining consistent immunostimulatory activity while minimizing unintended inflammatory signaling is essential. The improved protective efficacy observed with BECC438s, together with its structurally defined composition, supports further investigation of synthetic BECC adjuvants as next-generation vaccine platforms for human use.

Clinical Pa strains exhibit increased resistance to non-opsonic phagocytosis compared to other strains; the observed OPK activity is likely a decisive factor in reducing infection risk *in vivo* (44). This parallels clinical observations in CF patients, where Pa colonization correlates with impaired clearance due to capsule-mediated evasion of innate immunity. Thus, vaccines that elicit robust opsonic antibodies may overcome this barrier and confer meaningful protection prior to the establishment of chronic infection. Taken together, these findings suggest that serum immunoglobulins generated by L-PaF-based vaccines significantly contribute to opsonophagocytic killing of Pa, reinforcing their potential role in protective/preventative immunity. The superiority of IN immunization observed here is consistent with findings with other respiratory pathogens such as *Streptococcus pneumonia*e and *Bordetella pertussis*, where mucosal delivery enhances both systemic IgG and local IgA responses, leading to improved pathogen clearance from the lungs (50–52). Unlike IM immunization, which primarily induces circulating IgG, IN vaccination promotes immune imprinting at a mucosal site, facilitating recruitment of tissue-resident memory T cells and IgA-secreting plasma cells, which are key components for defense against inhaled pathogens. Moreover, mucosal adjuvants like BECC438 may amplify innate signaling pathways (as an agonist of TLR-4) within the respiratory tract, driving robust Th17 and IgA responses that complement opsonic IgG-mediated clearance (33). This contrasts with systemic adjuvants, which often skew toward Th1-based IgG responses without significant mucosal engagement. Enhanced lung clearance following IN immunization highlights the translational potential of mucosal vaccine strategies for preventing infection in high-risk populations, including individuals with CF or those susceptible to ventilator-associated pneumonia.

Effective protection against Pa also requires strong antibody‑mediated immunity. Th1-Th17 mediated response was assessed by quantifying key cytokines associated with these T-helper subsets, including IFN-γ, IL-2, IL-6, TNF-α, IL-22, and the Th17 signature cytokine IL-17A. IL-2 is a central regulator of T cell activation (53), and IL-6 helps the host immune system in the transition of innate to acquired (54). The literature suggests a positive association of IL-17A with mucosal vaccination in the form of memory B cell generation (55, 56). TNF-α and IFN-γ synergistically drive inflammation and immune activation by enhancing macrophage function, antigen presentation, and Th1-mediated pathogen clearance (57). IL-22 plays a protective role in acute Pa lung infections by promoting epithelial barrier maintenance, enhancing the production of antimicrobial proteins and chemokines, and moderating neutrophil accumulation in the lungs (58, 59). Multiple vaccine platforms, including PcrV, PopB, ExoS, and ExlA/L-PaF based formulations, have shown that inducing IL‑17A correlates with improved mucosal IgA, systemic IgG, and reduced lung colonization, often independent of opsonophagocytic antibody activity (18, 31, 43, 60). Live-attenuated and nanoparticle vaccines similarly demonstrated that IL‑17A is essential for protection, as its depletion abolishes protective immunity (61, 62). In our pre-challenged cytokine study, we have observed that the IN administration of L-PaF/ME with BECC438s or BECC438b vaccine elevated IL-17A responses compared to IM administration. These findings also align with previous studies on pneumococcal and pertussis vaccines, where systemic routes favor circulating IgG and Th1 responses, while mucosal routes induce mixed Th1/Th17 immunity with localized effector functions (63). A similar pattern was observed for experimental *Klebsiella pneumoniae* vaccines, where IL-17A-driven immunity is indispensable for defense against encapsulated strains (64). The robust Th1/Th17 cytokine milieu elicited by IN immunization likely underpins the superior lung clearance observed post-challenge. IL-17A and IL-22 enhance epithelial defense and neutrophil-mediated killing, complementing opsonic antibody functions. In our post-challenge cytokine study, IL-17A was elevated following IN immunization with L-PaF/ME/BECC438, correlated with reduced pulmonary bacterial burden compared to IM immunization. This suggests a functional link between Th17-driven immunity and enhanced pathogen clearance via IN administration, which is absent in IM immunized mice. This indicates that systemic vaccination fails to induce robust mucosal Th17 responses necessary for optimal lung protection. While TNF-α and IL-6 cytokines are indispensable for initiating inflammation, their lack of correlation with bacterial clearance in our study mirrors observations in severe pneumonia cases, where elevated TNF-α and IL-6 often predict pathology rather than protection (65, 66). The post-challenge cytokine response for the L-Paf/ME/BECC438 group vaccinated IN supports controlled cytokine mediated immunity to facilitate effective clearance during early bacterial infection. But PBS groups and L-Paf/ME/BECC470b or BECC470s vaccinated group, irrespective of route of administration, failed to maintain the proper cytokine homeostasis during infection and failed to provide optimal bacterial clearance. These distinct immune imprints may ultimately shape the magnitude and location of the protective response against respiratory pathogen challenge. The data support the hypothesis that mucosal immunization strategies leveraging Th17 responses are superior for combating respiratory pathogens like Pa. Altogether, our data support the use of L-PaF/ME/BECC438 as a promising vaccine candidate against Pa following IN vaccination. Nevertheless, while our findings support the IN route for Pa vaccination, there are limitations that must be addressed. First, long-term durability of the immune response and protection against diverse Pa strains were not evaluated here. Second, the impact of repeated dosing and potential mucosal tolerance requires further investigation. Future research should explore the integration of additional antigens to broaden coverage and assess the role of tissue-resident memory T cells in long-term protection. Systems immunology approaches may also help elucidate molecular paths activated by different routes and adjuvants, guiding rational vaccine design.

## Data Availability

The original contributions presented in the study are included in the article/**Supplementary Material**. Further inquiries can be directed to the corresponding author.
