# Supplementary material for: Comparative immunogenicity and protective efficacy of BECC438 and BECC470 adjuvants in a vaccine formulation against Pseudomonas aeruginosa
Source: Front Immunol. 2026 Sep 3;17:1908082. doi: 10.3389/fimmu.2026.1908082 (PMC13582523; doi:10.3389/fimmu.2026.1908082)
Supplement: Supplementary file 1 [file DataSheet1.docx]

**Supplemental figures for….**

**Comparative immunogenicity and protective efficacy of BECC438 and BECC470 adjuvants in a vaccine formulation against *Pseudomonas aeruginosa***

Prolay Halder^1,2^, Debaki Ranjan Howlader^1,2,#^, Satabdi Biswas^1,2^, Risha Haldar^1,2^, Suhrid Maiti^1,2^, Sayan Das^3^, Zackary K Dietz^1,2^, Ti Lu^1,2^, Sean K. Whittier^1,2^, Robert K Ernst^3^, William D Picking^1,2^, and Wendy L Picking^1,2^*

^1^Bond Life Sciences Center, University of Missouri, Columbia, MO, United States

^2^Department of Pathobiology & Integrative Biomedical Sciences, University of Missouri, Columbia, MO, United States

^3^Department of Microbial Pathogenesis, University of Maryland, Baltimore, MD, United States

^#^Present address: Division of Bacteriology, ICMR-National Institute for Research in Bacterial Infections, India

*Corresponding Author: Wendy L. Picking [wendy.picking@missouri.edu](mailto:wendy.picking@missouri.edu)

**Supplementary figures:**

**Supplementary Figure S1:**

A.i

A.ii

A.iii

B.i

B.ii

Biii

C.i

C.ii

C.iii

D.iii

D.ii

D.i

**Supplementary Figure S1: Lung cells secrete pro-inflammatory cytokines following *ex vivo* stimulation with PcrV and PopB.** Data for lung cells from intranasal (IN) immunized (**A.i-iii, B.i-iii**) and intramuscular (IM) immunized (**C.i-iii, D.i-iii**) pre-challenged mice following stimulation with PcrV (**A.i-iii, C.i-iii)** or PopB (**B.i-iii, D.i-iii**) are presented. Single cell suspensions of lung cell were treated with 10 μg/ml of either PcrV or PopB protein and incubated for 48 h at 37°C. Secretion of IFN-γ (**A.i, B.i, C.i, D.i**), IL-2 (**A.ii, B.ii, C.ii, D.ii**) and IL-22 (**A.iii, B.iii, C.iii, D.iii**) was determined for samples taken from IN or IM immunized pre-challenge mice. Cytokine levels were determined by Meso Scale Discovery (MSD) analysis as per manufacturer’s instructions and are presented as pg/ml/10^6^ cells. Data are plotted as actual values from individuals ± SD (n=5) in each group. Statistical significance was calculated by comparing the PBS group with their immunized counterparts using two-way ANOVA (Dunnett’s multiple comparison test). *p < 0.05, ***p < 0.001. Exact p values can be found in Supplementary Table.ST4.

**Supplementary Figure S2:**

A.i

A.ii

A.iii

B.i

B.ii

B.iii

C.i

C.ii

C.iii

D.i

D.ii

D.iii

**Supplementary Figure S2: Splenic cells secrete pro-inflammatory cytokines following *ex vivo* stimulation with PcrV and PopB**. Data for splenic cells from intranasal (IN) immunized (**A.i-iii, B.i-iii**) and intramuscular (IM) immunized (**C.i-iii, D.i-iii**) pre-challenged mice following stimulation with PcrV (**A.i-iii, C.i-iii**) or PopB (**B.i-iii, D.i-iii**) are presented. Single cell suspensions of spleens were treated with 10 μg/ml of either PcrV or PopB protein and incubated for 48 h at 37°C. Secretion of IFN-γ (**A.i, B.i, C.i, D.i**), IL-2 (**A.ii, B.ii, C.ii, D.ii**) and IL-22 (**A.iii, B.iii, C.iii, D.iii**) were determined for samples taken from IN or IM immunized pre-challenge mice. Cytokine levels were determined by Meso Scale Discovery (MSD) analysis as per manufacturer’s instructions and were presented as pg/ml/10^6^ cells. Data are plotted as actual values from individuals ± SD (n=5) in each group. Statistical significance was calculated by comparing the PBS group with their immunized counterparts using two-way ANOVA (Dunnett’s multiple comparison test). *p < 0.05, **p < 0.01, ***p < 0.001, ****p < 0.0001. Exact p values can be found in Supplementary Table. ST6.

**Supplementary Figure S3:**

A.i

A.ii

A.iii

B.iii

B.ii

B.i

**Supplementary Figure S3: Lung cells secrete pro-inflammatory cytokines following *ex vivo* cell culture 48h at 37°C without stimulation.** Data represents cytokine response of lung cells from intranasal (IN) immunized (**A.i-iii**) and intramuscular (IM) immunized (**B.i-iii**) pre-challenged mice in unstimulating condition. Single cell suspensions of lung tissues were maintained with complete RPMI media and incubated for 48 h at 37°C. Secretion of IL-17A (**A.i, B.i**), IL-6 (**A.ii, B.ii**) and TNF-α (**A.iii, B.iii**) for samples prepared from IN or IM immunized pre-challenge mice. Cytokine levels were determined by Meso Scale Discovery (MSD) analysis as per manufacturer’s instructions and were presented as pg/ml/10^6^ cells. Data are plotted as actual values from individuals ± SD (n=5) in each group. Statistical significance was calculated by comparing the PBS group with their immunized counterparts using two-way ANOVA (Dunnett’s multiple comparison test). *p < 0.05, **p < 0.01, ***p < 0.001. Exact p values can be found in Supplementary Table.ST10.
