# Supplementary material for: Comparative immunogenicity and protective efficacy of BECC438 and BECC470 adjuvants in a vaccine formulation against Pseudomonas aeruginosa
Source: Front Immunol. 2026 Sep 3;17:1908082. doi: 10.3389/fimmu.2026.1908082 (PMC13582523; doi:10.3389/fimmu.2026.1908082)
Supplement: Supplementary file 2 [file SupplementaryFile1.docx]

**Supplementary Tables:**

**Supplementary Table ST1:** Statistical comparison of OPK activity of intranasal (IN) immunized L-PaF formulation groups to PBS group of Figure: 4.

| **Comparison** | **Summary** | **p-value** |
| --- | --- | --- |
| PBS vs. Serum Control | ns | 0.5483 |
| PBS vs. L-PaF BECC 438b ME | *** | 0.0001 |
| PBS vs. L-PaF BECC 438s ME | **** | <0.0001 |
| PBS vs. L-PaF BECC 470b ME | *** | 0.0002 |
| PBS vs. L-PaF BECC 470s ME | *** | 0.0001 |

**Supplementary Table ST2:** Statistical comparison of OPK activity of intramuscular (IM) immunized L-PaF formulation groups to PBS group of Figure: 4.

| **Comparison** | **Summary** | **p-value** |
| --- | --- | --- |
| PBS vs. Serum Control | ns | 0.3458 |
| PBS vs. L-PaF BECC 438b ME | **** | <0.0001 |
| PBS vs. L-PaF BECC 438s ME | **** | <0.0001 |
| PBS vs. L-PaF BECC 470b ME | **** | <0.0001 |
| PBS vs. L-PaF BECC 470s ME | **** | <0.0001 |

**Supplementary Table ST3.** Statistical comparison of cytokines (IL-17A, IL-6, TNF- α) response secreted from lung cells of intranasal (IN) immunized and intramuscular (IM) immunized L-PaF formulation groups to PBS group after stimulation with PcrV and PopB of Figure: 5.

| **Route of Vaccination** | **Stimulation** | **Comparison** | **IL-17A** | | **IL-6** | | **TNF-α** | |
| --- | --- | --- | --- | --- | --- | --- | --- | --- |
| Intranasal Vaccinated | PcrV |  | p-value | Summary | p-value | Summary | p-value | Summary |
|  |  | PBS vs. L-PaF BECC 438b ME | ** | 0.0018 | ns | 0.0648 | ns | 0.0507 |
|  |  | PBS vs. L-PaF BECC 438s ME | ** | 0.0041 | ns | 0.0751 | ns | 0.0542 |
|  |  | PBS vs. L-PaF BECC 470b ME | ** | 0.0022 | ns | 0.1595 | ns | 0.3312 |
|  |  | PBS vs. L-PaF BECC 470s ME | ns | 0.4475 | ns | 0.3564 | ns | 0.9815 |
|  | PopB | **Comparison** | **IL-17A** | | **IL-6** | | **TNF-α** | |
|  |  |  | p-value | Summary | p-value | Summary | p-value | Summary |
|  |  | PBS vs. L-PaF BECC 438b ME | **** | <0.0001 | * | 0.0386 | * | 0.0314 |
|  |  | PBS vs. L-PaF BECC 438s ME | **** | <0.0001 | ns | 0.3491 | ns | 0.0794 |
|  |  | PBS vs. L-PaF BECC 470b ME | *** | 0.0003 | ns | 0.5077 | ns | 0.1409 |
|  |  | PBS vs. L-PaF BECC 470s ME | *** | 0.0008 | ns | 0.6079 | ns | 0.6126 |
| Intramuscular Vaccinated | PcrV | **Comparison** | **IL-17A** | | **IL-6** | | **TNF-α** | |
|  |  |  | p-value | Summary | p-value | Summary | p-value | Summary |
|  |  | PBS vs. L-PaF BECC 438b ME | ns | 0.8699 | ns | 0.7038 | * | 0.0124 |
|  |  | PBS vs. L-PaF BECC 438s ME | ns | 0.4075 | ns | 0.5216 | ns | 0.3223 |
|  |  | PBS vs. L-PaF BECC 470b ME | ns | 0.9999 | ns | 0.9158 | ns | 0.9958 |
|  |  | PBS vs. L-PaF BECC 470s ME | ns | >0.9999 | ns | 0.9143 | ns | 0.9471 |
|  | PopB | **Comparison** | **IL-17A** | | **IL-6** | | **TNF-α** | |
|  |  |  | p-value | Summary | p-value | Summary | p-value | Summary |
|  |  | PBS vs. L-PaF BECC 438b ME | * | 0.0313 | ns | 0.0522 | * | 0.0105 |
|  |  | PBS vs. L-PaF BECC 438s ME | ns | 0.2205 | * | 0.0154 | * | 0.0239 |
|  |  | PBS vs. L-PaF BECC 470b ME | ns | 0.9393 | ns | 0.1171 | ** | 0.0098 |
|  |  | PBS vs. L-PaF BECC 470s ME | ns | 0.9999 | ns | 0.9192 | ns | 0.2365 |

**Supplementary Table ST4.** Statistical comparison of cytokines (IFN-γ, IL-2, IL-22) response secreted from lung cells of intranasal (IN) immunized and intramuscular (IM) immunized L-PaF formulation groups to PBS group after stimulation with PcrV and PopB of Supplementary Figure. S1.

| **Route of Vaccination** | **Stimulation** | **Comparison** | **IFN-γ** | | **IL-2** | | **IL-22** | |
| --- | --- | --- | --- | --- | --- | --- | --- | --- |
| Intranasal Vaccinated | PcrV |  | p-value | Summary | p-value | Summary | p-value | Summary |
|  |  | PBS vs. L-PaF BECC 438b ME | ns | 0.1735 | * | 0.0218 | ns | 0.4719 |
|  |  | PBS vs. L-PaF BECC 438s ME | ns | 0.1913 | * | 0.0259 | ns | 0.0805 |
|  |  | PBS vs. L-PaF BECC 470b ME | ns | 0.2631 | *** | 0.0003 | ns | 0.1257 |
|  |  | PBS vs. L-PaF BECC 470s ME | ns | 0.9909 | ns | 0.3686 | ns | 0.9808 |
|  | PopB | **Comparison** | **IFN-γ** | | **IL-2** | | **IL-22** | |
|  |  |  | p-value | Summary | p-value | Summary | p-value | Summary |
|  |  | PBS vs. L-PaF BECC 438b ME | * | 0.0406 | ns | 0.4756 | ns | 0.0888 |
|  |  | PBS vs. L-PaF BECC 438s ME | ns | 0.0561 | ns | 0.3575 | * | 0.0301 |
|  |  | PBS vs. L-PaF BECC 470b ME | ns | 0.0544 | ns | 0.1446 | ns | 0.1559 |
|  |  | PBS vs. L-PaF BECC 470s ME | ns | 0.3564 | ns | 0.3688 | ns | 0.7263 |
| Intramuscular Vaccinated | PcrV | **Comparison** | **IFN-γ** | | **IL-2** | | **IL-22** | |
|  |  |  | p-value | Summary | p-value | Summary | p-value | Summary |
|  |  | PBS vs. L-PaF BECC 438b ME | ns | 0.5141 | ns | 0.8104 | ns | 0.1179 |
|  |  | PBS vs. L-PaF BECC 438s ME | ns | 0.9567 | ns | 0.4320 | ns | 0.9865 |
|  |  | PBS vs. L-PaF BECC 470b ME | ns | 0.8475 | ns | 0.9966 | ns | >0.9999 |
|  |  | PBS vs. L-PaF BECC 470s ME | ns | 0.9965 | ns | >0.9999 | ns | >0.9999 |
|  | PopB | **Comparison** | **IFN-γ** | | **IL-2** | | **IL-22** | |
|  |  |  | p-value | Summary | p-value | Summary | p-value | Summary |
|  |  | PBS vs. L-PaF BECC 438b ME | * | 0.0306 | ns | 0.1682 | ns | 0.5037 |
|  |  | PBS vs. L-PaF BECC 438s ME | * | 0.0306 | * | 0.0335 | ns | 0.0765 |
|  |  | PBS vs. L-PaF BECC 470b ME | ns | 0.4657 | ns | 0.0688 | ns | 0.1062 |
|  |  | PBS vs. L-PaF BECC 470s ME | ns | 0.9977 | ns | 0.2971 | ns | 0.9996 |

**Supplementary Table ST5.** Statistical comparison of cytokines (IL-17A, IL-6, TNF- α) response secreted from splenic cells of intranasal (IN) immunized and intramuscular (IM) immunized L-PaF formulation groups to PBS group after stimulation with PcrV and PopB of Figure: 6.

| **Route of Vaccination** | **Stimulation** | **Comparison** | **IL-17A** | | **IL-6** | | **TNF-α** | |
| --- | --- | --- | --- | --- | --- | --- | --- | --- |
| Intranasal Vaccinated | PcrV |  | p-value | Summary | p-value | Summary | p-value | Summary |
|  |  | PBS vs. L-PaF BECC 438b ME | ns | 0.7552 | ns | 0.4732 | ns | 0.8167 |
|  |  | PBS vs. L-PaF BECC 438s ME | ns | 0.0659 | ns | 0.1000 | * | 0.0439 |
|  |  | PBS vs. L-PaF BECC 470b ME | ns | 0.5400 | ns | 0.3124 | ns | 0.5880 |
|  |  | PBS vs. L-PaF BECC 470s ME | ns | 0.9200 | ns | 0.5083 | ns | 0.7476 |
|  | PopB | **Comparison** | **IL-17A** | | **IL-6** | | **TNF-α** | |
|  |  |  | p-value | Summary | p-value | Summary | p-value | Summary |
|  |  | PBS vs. L-PaF BECC 438b ME | ** | 0.0028 | ns | 0.1513 | ns | 0.1735 |
|  |  | PBS vs. L-PaF BECC 438s ME | ** | 0.0057 | ns | 0.1215 | ** | 0.0080 |
|  |  | PBS vs. L-PaF BECC 470b ME | ** | 0.0061 | * | 0.0363 | * | 0.0350 |
|  |  | PBS vs. L-PaF BECC 470s ME | ns | 0.0897 | ns | 0.1874 | ns | 0.0716 |
| Intramuscular Vaccinated | PcrV | **Comparison** | **IL-17A** | | **IL-6** | | **TNF-α** | |
|  |  |  | p-value | Summary | p-value | Summary | p-value | Summary |
|  |  | PBS vs. L-PaF BECC 438b ME | ns | 0.2736 | ns | 0.5954 | ns | 0.8659 |
|  |  | PBS vs. L-PaF BECC 438s ME | ns | 0.9964 | * | 0.0464 | * | 0.0311 |
|  |  | PBS vs. L-PaF BECC 470b ME | ns | >0.9999 | ns | 0.9988 | ns | 0.9449 |
|  |  | PBS vs. L-PaF BECC 470s ME | ns | >0.9999 | ns | 0.9855 | ns | 0.9894 |
|  | PopB | **Comparison** | **IL-17A** | | **IL-6** | | **TNF-α** | |
|  |  |  | p-value | Summary | p-value | Summary | p-value | Summary |
|  |  | PBS vs. L-PaF BECC 438b ME | ns | 0.0874 | ** | 0.0091 | ns | 0.9986 |
|  |  | PBS vs. L-PaF BECC 438s ME | ns | 0.6008 | ns | 0.0939 | ns | >0.9999 |
|  |  | PBS vs. L-PaF BECC 470b ME | ns | >0.9999 | ns | 0.5871 | ns | 0.6037 |
|  |  | PBS vs. L-PaF BECC 470s ME | ns | 0.9538 | ns | 0.8251 | ** | 0.0086 |

**Supplementary Table ST6.** Statistical comparison of cytokines (IFN-γ, IL-2, IL-22) response secreted from Splenic cells of intranasal (IN) immunized and intramuscular (IM) immunized L-PaF formulation groups to PBS group after stimulation with PcrV and PopB of Supplementary Figure. S2.

| **Route of Vaccination** | **Stimulation** | **Comparison** | **IFN-γ** | | **IL-2** | | **IL-22** | |
| --- | --- | --- | --- | --- | --- | --- | --- | --- |
| Intranasal Vaccinated | PcrV |  | p-value | Summary | p-value | Summary | p-value | Summary |
|  |  | PBS vs. L-PaF BECC 438b ME | ns | 0.9861 | ns | 0.2319 | ns | 0.9975 |
|  |  | PBS vs. L-PaF BECC 438s ME | ns | 0.3397 | ns | 0.6226 | ns | 0.2772 |
|  |  | PBS vs. L-PaF BECC 470b ME | ns | 0.6934 | *** | 0.0009 | ns | 0.9946 |
|  |  | PBS vs. L-PaF BECC 470s ME | ns | 0.9023 | ns | 0.9746 | ns | 0.9989 |
|  | PopB | **Comparison** | **IFN-γ** | | **IL-2** | | **IL-22** | |
|  |  |  | p-value | Summary | p-value | Summary | p-value | Summary |
|  |  | PBS vs. L-PaF BECC 438b ME | * | 0.0321 | ns | 0.2253 | ns | 0.5879 |
|  |  | PBS vs. L-PaF BECC 438s ME | ns | 0.0572 | ns | 0.2819 | ns | 0.2276 |
|  |  | PBS vs. L-PaF BECC 470b ME | * | 0.0177 | ns | 0.1094 | ns | 0.3616 |
|  |  | PBS vs. L-PaF BECC 470s ME | * | 0.0128 | ns | 0.9664 | ns | 0.8486 |
| Intramuscular Vaccinated | PcrV | **Comparison** | **IFN-γ** | | **IL-2** | | **IL-22** | |
|  |  |  | p-value | Summary | p-value | Summary | p-value | Summary |
|  |  | PBS vs. L-PaF BECC 438b ME | ns | 0.4648 | ns | 0.5426 | ns | 0.3426 |
|  |  | PBS vs. L-PaF BECC 438s ME | ns | 0.0895 | ns | 0.6701 | ns | 0.9985 |
|  |  | PBS vs. L-PaF BECC 470b ME | ns | >0.9999 | ns | 0.7148 | ns | >0.9999 |
|  |  | PBS vs. L-PaF BECC 470s ME | ns | 0.9472 | ns | 0.9961 | ns | >0.9999 |
|  | PopB | **Comparison** | **IFN-γ** | | **IL-2** | | **IL-22** | |
|  |  |  | p-value | Summary | p-value | Summary | p-value | Summary |
|  |  | PBS vs. L-PaF BECC 438b ME | **** | <0.0001 | * | 0.0262 | ** | 0.0017 |
|  |  | PBS vs. L-PaF BECC 438s ME | **** | <0.0001 | *** | 0.0004 | *** | 0.0004 |
|  |  | PBS vs. L-PaF BECC 470b ME | * | 0.0347 | ns | 0.2870 | ns | 0.9960 |
|  |  | PBS vs. L-PaF BECC 470s ME | ns | 0.0576 | **** | <0.0001 | ns | >0.9999 |

**Supplementary Table ST7.** Statistical comparison of relative lung burdens of intranasal (IN) and intramuscular (IM) immunized L-PaF formulation groups to PBS group of Figure: 7.

| **Comparison** | **Summary** | **p-value** |
| --- | --- | --- |
| **Intranasal (IN) immunized** | | |
| PBS vs. L-PaF BECC 438b ME | ** | 0.0021 |
| PBS vs. L-PaF BECC 438s ME | **** | <0.0001 |
| PBS vs. L-PaF BECC 470b ME | ns | 0.0880 |
| PBS vs. L-PaF BECC 470s ME | ns | 0.4312 |
| **Intramuscular (IM) immunized** | | |
| PBS vs. L-PaF BECC 438b ME | ns | 0.3285 |
| PBS vs. L-PaF BECC 438s ME | ns | 0.0825 |
| PBS vs. L-PaF BECC 470b ME | ns | 0.2530 |
| PBS vs. L-PaF BECC 470s ME | ns | 0.7434 |

**Supplementary Table ST8:** Exact % reduction of lung burden of intranasal (IN) and intramuscular (IM) immunized L-PaF formulation groups to PBS group of Figure: 7.

| Group | IN in-vivo % reduction comparison to PBS group | IM in-vivo % reduction comparison to PBS group |
| --- | --- | --- |
| L-PaF/ME/BECC438b | 97.83050847 | 80.47606989 |
| L-PaF/ME/BECC438s | 99.81355932 | 97.43479362 |
| L-PaF/ME/BECC470b | 90.80508475 | 93.73259053 |
| L-PaF/ME/BECC470s | 36.3559322 | 54.21878957 |

**Supplementary Table ST9.** Statistical comparison of cytokines (IL-17A, IL-6, TNF-α) response secreted from lung cells of intranasal (IN) immunized and intramuscular (IM) immunized L-PaF formulation groups to PBS group after post-challenge with Pa, of Figure: 8.

| **Route of Challenge** | **Route of vaccination** | **Comparison** | **IL-17A** | | **IL-6** | | **TNF-α** | |
| --- | --- | --- | --- | --- | --- | --- | --- | --- |
| Lung Post-challenge with Pa | Intranasal Vaccinated |  | p-value | Summary | p-value | Summary | p-value | Summary |
|  |  | PBS vs. L-PaF BECC 438b ME | ns | 0.9975 | ns | 0.2716 | ns | 0.9286 |
|  |  | PBS vs. L-PaF BECC 438s ME | ns | 0.2898 | ns | >0.9999 | ns | 0.9980 |
|  |  | PBS vs. L-PaF BECC 470b ME | ns | 0.9262 | ns | 0.5032 | ns | 0.1789 |
|  |  | PBS vs. L-PaF BECC 470s ME | ns | >0.9999 | ns | 0.2619 | ns | 0.4190 |
|  | Intramuscular Vaccinated | **Comparison** | **IL-17A** | | **IL-6** | | **TNF-α** | |
|  |  |  | p-value | Summary | p-value | Summary | p-value | Summary |
|  |  | PBS vs. L-PaF BECC 438b ME | ns | 0.4481 | ns | 0.9947 | ns | 0.2315 |
|  |  | PBS vs. L-PaF BECC 438s ME | ns | 0.4236 | ns | 0.2237 | * | 0.0328 |
|  |  | PBS vs. L-PaF BECC 470b ME | ns | 0.7845 | ns | 0.9346 | ns | 0.1543 |
|  |  | PBS vs. L-PaF BECC 470s ME | ns | 0.5686 | ns | 0.1509 | ns | 0.2294 |

**Supplementary Table ST10.** Statistical comparison of cytokines (IL-17A, IL-6, TNF-α) response secreted from lung cells of intranasal (IN) immunized and intramuscular (IM) immunized L-PaF formulation groups to PBS group of pre-challenge samples, of Supplementary Figure. S3.

| **Route of vaccination** | **Comparison** | **IL-17A** | | **IL-6** | | **TNF-α** | |
| --- | --- | --- | --- | --- | --- | --- | --- |
| Intranasal Vaccinated |  | p-value | Summary | p-value | Summary | p-value | Summary |
|  | PBS vs. L-PaF BECC 438b ME | ** | 0.0029 | * | 0.0324 | ns | 0.2132 |
|  | PBS vs. L-PaF BECC 438s ME | ** | 0.0023 | * | 0.0362 | ns | 0.1094 |
|  | PBS vs. L-PaF BECC 470b ME | * | 0.0226 | ns | 0.1111 | ns | 0.6042 |
|  | PBS vs. L-PaF BECC 470s ME | ns | 0.7117 | ns | 0.6273 | ns | 0.9957 |
| Intramuscular Vaccinated | **Comparison** | **IL-17A** | | **IL-6** | | **TNF-α** | |
|  |  | p-value | Summary | p-value | Summary | p-value | Summary |
|  | PBS vs. L-PaF BECC 438b ME | ns | 0.8989 | ** | 0.0087 | ** | 0.0050 |
|  | PBS vs. L-PaF BECC 438s ME | ns | 0.9570 | *** | 0.0009 | * | 0.0221 |
|  | PBS vs. L-PaF BECC 470b ME | ns | 0.8419 | ns | >0.9999 | *** | 0.0002 |
|  | PBS vs. L-PaF BECC 470s ME | ns | 0.3047 | ** | 0.0053 | *** | 0.0003 |

**Supplementary Table ST11.** Exact fold-change values & Log_2_ values of Fold Change of different cytokines between pre-challenge and post-challenge unstimulated lung cytokines were evaluated for IN immunization and IM immunization, of Figure: 8.

| **Group**  **(**IN Immunization**)** | **Pre IL-17A unstimulated** | **Post IL-17A unstimulated** | **Fold Change** | **Log_2_ value of Fold Change** |
| --- | --- | --- | --- | --- |
| PBS | 7.172937 | 2079.885 | 289.9627886 | 8.179 |
| L-PaF/BECC 438b/ ME | 1951.62 | 1816.6 | 0.930816442 | -0.103 |
| L-PaF/BECC 438s/ ME | 2008.059 | 3964.955 | 1.974520782 | 0.981 |
| L-PaF/BECC 470b/ ME | 1480.812 | 2754.786 | 1.860320949 | 0.895 |
| L-PaF/BECC 470s/ ME | 484.323 | 2021.682 | 4.174236761 | 2.061 |
| **Group**  **(**IN Immunization**)** | **Pre IL-6 unstimulated** | **Post IL-6 unstimulated** | **Fold Change** | **Log_2_ value of Fold Change** |
| PBS | 238.5968 | 5747.426386 | 24.08845076 | 4.59 |
| L-PaF/BECC 438b/ ME | 2128.788 | 3126.597759 | 1.468722084 | 0.554 |
| L-PaF/BECC 438s/ ME | 2093.226 | 5843.007679 | 2.791389055 | 1.48 |
| L-PaF/BECC 470b/ ME | 1713.213 | 3775.069797 | 2.203503139 | 1.139 |
| L-PaF/BECC 470s/ ME | 970.2235 | 3091.812397 | 3.186701342 | 1.672 |
| **Group**  **(**IN Immunization**)** | **Pre** **TNF-α**  **unstimulated** | **Post TNF-α unstimulated** | **Fold Change** | **Log_2_ value of Fold Change** |
| PBS | 82.08663399 | 764.8230971 | 9.317267136 | 3.219 |
| L-PaF/BECC 438b/ ME | 243.9814124 | 659.6169271 | 2.703554015 | 1.434 |
| L-PaF/BECC 438s/ ME | 276.4958013 | 725.9200688 | 2.625428905 | 1.392 |
| L-PaF/BECC 470b/ ME | 181.1258723 | 416.5568515 | 2.299819712 | 1.201 |
| L-PaF/BECC 470s/ ME | 58.72840824 | 511.7264316 | 8.713439491 | 3.123 |

| **Group**  **(**IM Immunization**)** | **Pre IL-17A unstimulated** | **Post IL-17A unstimulated** | **Fold Change** | **Log_2_ value of Fold Change** |
| --- | --- | --- | --- | --- |
| PBS | 2.4 | 712.4 | 296.8333333 | 8.213 |
| L-PaF/BECC 438b/ ME | 142.6 | 348.6 | 2.444600281 | 1.289 |
| L-PaF/BECC 438s/ ME | 109.8 | 337.8 | 3.076502732 | 1.621 |
| L-PaF/BECC 470b/ ME | 45.4 | 482.8 | 10.63436123 | 3.410 |
| L-PaF/BECC 470s/ ME | 349.4 | 398 | 1.139095592 | 0.187 |
| **Group**  **(**IM Immunization**)** | **Pre IL-6 unstimulated** | **Post IL-6 unstimulated** | **Fold Change** | **Log_2_ value of Fold Change** |
| PBS | 1527.4 | 5379.4 | 3.521932696 | 1.816 |
| L-PaF/BECC 438b/ ME | 11739 | 5832.2 | 0.496822557 | -1.00 |
| L-PaF/BECC 438s/ ME | 14937.6 | 2462.2 | 0.164832369 | -2.600 |
| L-PaF/BECC 470b/ ME | 1350 | 4460.8 | 3.304296296 | 1.724 |
| L-PaF/BECC 470s/ ME | 12413.4 | 2101.2 | 0.169268694 | -2.562 |
| **Group**  **(**IM Immunization**)** | **Pre TNF-α**  **unstimulated** | **Post TNF-α unstimulated** | **Fold Change** | **Log_2_ value of Fold Change** |
| PBS | 1276.4 | 1848.4 | 1.448135381 | 0.534 |
| L-PaF/BECC 438b/ ME | 653.4 | 917 | 1.403428222 | 0.488 |
| L-PaF/BECC 438s/ ME | 771.4 | 384.6 | 0.498574021 | -1.004 |
| L-PaF/BECC 470b/ ME | 406.8 | 796.4 | 1.957718781 | 0.969 |
| L-PaF/BECC 470s/ ME | 436.6 | 914.2 | 2.093907467 | 1.066 |

**Supplementary Table ST12.** Correlation between Log_2_ Fold change of IL-17A (Pre- & Post-infection unstimulated ex-vivo lung cells secreted IL-17A) and *in-vivo* % reduction of bacterial burden in lung. Correlation assays were done in GraphPad and Pearson r was determined, of Figure. 9.

| Log_2_ Fold change of IL-17A (Pre- & Post-infection unstimulated)  vs.  *in-vivo* % reduction of bacterial burden | |
| --- | --- |
| **Pearson r** |  |
| r | -0.8161 |
| 95% confidence interval | -0.9550 to -0.3835 |
| R squared | 0.6660 |
| **P value** |  |
| P (two-tailed) | 0.0040 |
| P value summary | ** |
| Significant? (alpha = 0.05) | Yes |
